# Supplementary material for: COZOID: contact zone identifier for visual analysis of protein-protein interactions
Source: BMC Bioinformatics. 2018 Apr 6;19:125. doi: 10.1186/s12859-018-2113-6 (PMC5889581; doi:10.1186/s12859-018-2113-6)
Supplement: Supplementary file 2 — Software build. Executable binary file of the software tool. (ZIP 118,784 kb) [file 12859_2018_2113_MOESM2_ESM.zip › cozoid/platform/docs/testng-6.5.1-javadoc.zip!/index-all.html]

 
 
 
 
 
 
Index (TestNG)
 

 

 

 
function windowTitle()
{
    if (location.href.indexOf('is-external=true') == -1) {
        parent.document.title="Index (TestNG)";
    }
}
 
 
 

 

 
 


 
   
  
 
 
 
   
 
   
          Overview     
        Package   
        Class   
          Tree     
          Deprecated     
       Index    
          Help     
   
 
 
  
 
 
 

 
  
 PREV 
 NEXT  
  
    FRAMES     
   NO FRAMES     
  
    All Classes  ');
  }
  //-->
 
 
    All Classes  
 


  
 
 
  
 

 A   B   C   D   E   F   G   H   I   J   L   M   N   O   P   R   S   T   U   V   W   X   
    
 A  
 
   actOnResult(int, boolean)   - 
Method in class org.testng. TestNGAntTask 
  
   addClassfileset(FileSet)   - 
Method in class org.testng. TestNGAntTask 
  
   addClassLoader(ClassLoader)   - 
Method in class org.testng. TestNG 
 Add a class loader to the searchable loaders.
   addConfiguredPropertySet(PropertySet)   - 
Method in class org.testng. TestNGAntTask 
 Add the referenced property set as system properties for the TestNG JVM.
   addConfiguredReporter(ReporterConfig)   - 
Method in class org.testng. TestNGAntTask 
  
   addEnv(Environment.Variable)   - 
Method in class org.testng. TestNGAntTask 
 Adds an environment variable; used when forking.
   addExcludedGroup(String)   - 
Method in class org.testng.xml. XmlSuite 
  
   addExcludedGroup(String)   - 
Method in class org.testng.xml. XmlTest 
  
   addExecutionListener(IExecutionListener)   - 
Method in class org.testng. TestNG 
  
   addFailedButWithinSuccessPercentageTest(ITestNGMethod, ITestResult)   - 
Method in class org.testng. TestRunner 
  
   addFailedInvocationNumber(int)   - 
Method in interface org.testng. ITestNGMethod 
 The list of invocation numbers that failed, which is only applicable for
 methods that have a data provider.
   addFailedTest(ITestNGMethod, ITestResult)   - 
Method in class org.testng. TestRunner 
  
   addGuiceModule(Class<? extends Module>, Module)   - 
Method in interface org.testng. ITestContext 
  
   addGuiceModule(Class<? extends Module>, Module)   - 
Method in class org.testng. TestRunner 
  
   addIncludedGroup(String)   - 
Method in class org.testng.xml. XmlSuite 
  
   addIncludedGroup(String)   - 
Method in class org.testng.xml. XmlTest 
  
   addInjector(List<Module>, Injector)   - 
Method in interface org.testng. ITestContext 
  
   addInjector(List<Module>, Injector)   - 
Method in class org.testng. TestRunner 
  
   addInstance(Object)   - 
Method in interface org.testng. IClass 
  
   addInvokedMethod(InvokedMethod)   - 
Method in class org.testng. TestRunner 
  
   addInvokedMethodListener(IInvokedMethodListener)   - 
Method in class org.testng. TestNG 
  
   addListener(ITestNGListener)   - 
Method in interface org.testng. ISuite 
  
   addListener(ISuiteListener)   - 
Method in class org.testng. SuiteRunner 
 Registers ISuiteListeners interested in reporting the result of the current
 suite.
   addListener(ITestNGListener)   - 
Method in class org.testng. SuiteRunner 
  
   addListener(Object)   - 
Method in class org.testng. TestNG 
  
   addListener(IInvokedMethodListener)   - 
Method in class org.testng. TestNG 
  
   addListener(ISuiteListener)   - 
Method in class org.testng. TestNG 
  
   addListener(ITestListener)   - 
Method in class org.testng. TestNG 
  
   addListener(IReporter)   - 
Method in class org.testng. TestNG 
  
   addListener(Object)   - 
Method in class org.testng. TestRunner 
  
   addListener(String)   - 
Method in class org.testng.xml. XmlSuite 
  
   addMetaGroup(String, List<String>)   - 
Method in class org.testng.xml. XmlTest 
  
   addMethodDependedUpon(String)   - 
Method in interface org.testng. ITestNGMethod 
  
   addMethodSelector(String, int)   - 
Method in class org.testng. TestNG 
  
   addParameter(String, String)   - 
Method in class org.testng.xml. XmlTest 
  
   addPassedTest(ITestNGMethod, ITestResult)   - 
Method in class org.testng. TestRunner 
  
   addProperty(ReporterConfig.Property)   - 
Method in class org.testng. ReporterConfig 
  
   addResult(ITestResult, ITestNGMethod)   - 
Method in interface org.testng. IResultMap 
  
   addSkippedTest(ITestNGMethod, ITestResult)   - 
Method in class org.testng. TestRunner 
  
   addSysproperty(Environment.Variable)   - 
Method in class org.testng. TestNGAntTask 
  
   addTestListener(ITestListener)   - 
Method in class org.testng. TestRunner 
  
   addXmlfileset(FileSet)   - 
Method in class org.testng. TestNGAntTask 
  
   AfterClass   - Annotation Type in  org.testng.annotations      AfterGroups   - Annotation Type in  org.testng.annotations      afterInvocation(IInvokedMethod, ITestResult)   - 
Method in interface org.testng. IInvokedMethodListener 
  
   afterInvocation(IInvokedMethod, ITestResult, ITestContext)   - 
Method in interface org.testng. IInvokedMethodListener2 
 To be implemented if the method needs a handle to contextual information.
   afterInvocation(IInvokedMethod, ITestResult)   - 
Method in class org.testng. SuiteRunner 
  
   AfterMethod   - Annotation Type in  org.testng.annotations      AfterSuite   - Annotation Type in  org.testng.annotations      AfterTest   - Annotation Type in  org.testng.annotations      Assert   - Class in  org.testng  Assertion tool class.   Assert()   - 
Constructor for class org.testng. Assert 
 Protect constructor since it is a static only class
   assertDirectory(File, String)   - 
Static method in class org.testng. FileAssert 
 Asserts that a  tstvalue  is a proper directory.
   assertDirectory(File)   - 
Static method in class org.testng. FileAssert 
  
   assertEquals(Object, Object, String)   - 
Static method in class org.testng. Assert 
 Asserts that two objects are equal.
   assertEquals(Object, Object)   - 
Static method in class org.testng. Assert 
 Asserts that two objects are equal.
   assertEquals(String, String, String)   - 
Static method in class org.testng. Assert 
 Asserts that two Strings are equal.
   assertEquals(String, String)   - 
Static method in class org.testng. Assert 
 Asserts that two Strings are equal.
   assertEquals(double, double, double, String)   - 
Static method in class org.testng. Assert 
 Asserts that two doubles are equal concerning a delta.
   assertEquals(double, double, double)   - 
Static method in class org.testng. Assert 
 Asserts that two doubles are equal concerning a delta.
   assertEquals(float, float, float, String)   - 
Static method in class org.testng. Assert 
 Asserts that two floats are equal concerning a delta.
   assertEquals(float, float, float)   - 
Static method in class org.testng. Assert 
 Asserts that two floats are equal concerning a delta.
   assertEquals(long, long, String)   - 
Static method in class org.testng. Assert 
 Asserts that two longs are equal.
   assertEquals(long, long)   - 
Static method in class org.testng. Assert 
 Asserts that two longs are equal.
   assertEquals(boolean, boolean, String)   - 
Static method in class org.testng. Assert 
 Asserts that two booleans are equal.
   assertEquals(boolean, boolean)   - 
Static method in class org.testng. Assert 
 Asserts that two booleans are equal.
   assertEquals(byte, byte, String)   - 
Static method in class org.testng. Assert 
 Asserts that two bytes are equal.
   assertEquals(byte, byte)   - 
Static method in class org.testng. Assert 
 Asserts that two bytes are equal.
   assertEquals(char, char, String)   - 
Static method in class org.testng. Assert 
 Asserts that two chars are equal.
   assertEquals(char, char)   - 
Static method in class org.testng. Assert 
 Asserts that two chars are equal.
   assertEquals(short, short, String)   - 
Static method in class org.testng. Assert 
 Asserts that two shorts are equal.
   assertEquals(short, short)   - 
Static method in class org.testng. Assert 
 Asserts that two shorts are equal.
   assertEquals(int, int, String)   - 
Static method in class org.testng. Assert 
 Asserts that two ints are equal.
   assertEquals(int, int)   - 
Static method in class org.testng. Assert 
 Asserts that two ints are equal.
   assertEquals(Collection, Collection)   - 
Static method in class org.testng. Assert 
 Asserts that two collections contain the same elements in the same order.
   assertEquals(Collection, Collection, String)   - 
Static method in class org.testng. Assert 
 Asserts that two collections contain the same elements in the same order.
   assertEquals(Object[], Object[], String)   - 
Static method in class org.testng. Assert 
 Asserts that two arrays contain the same elements in the same order.
   assertEquals(Object[], Object[])   - 
Static method in class org.testng. Assert 
 Asserts that two arrays contain the same elements in the same order.
   assertEquals(byte[], byte[])   - 
Static method in class org.testng. Assert 
 Asserts that two arrays contain the same elements in the same order.
   assertEquals(byte[], byte[], String)   - 
Static method in class org.testng. Assert 
 Asserts that two arrays contain the same elements in the same order.
   assertEquals(Set, Set)   - 
Static method in class org.testng. Assert 
 Asserts that two sets are equal.
   assertEquals(Set<?>, Set<?>, String)   - 
Static method in class org.testng. Assert 
 Assert set equals
   assertEquals(Map<?, ?>, Map<?, ?>)   - 
Static method in class org.testng. Assert 
 Asserts that two maps are equal.
   assertEquals(String, Object, Object)   - 
Static method in class org.testng. AssertJUnit 
 Asserts that two objects are equal.
   assertEquals(Object, Object)   - 
Static method in class org.testng. AssertJUnit 
 Asserts that two objects are equal.
   assertEquals(String, String, String)   - 
Static method in class org.testng. AssertJUnit 
 Asserts that two Strings are equal.
   assertEquals(String, String)   - 
Static method in class org.testng. AssertJUnit 
 Asserts that two Strings are equal.
   assertEquals(String, double, double, double)   - 
Static method in class org.testng. AssertJUnit 
 Asserts that two doubles are equal concerning a delta.
   assertEquals(double, double, double)   - 
Static method in class org.testng. AssertJUnit 
 Asserts that two doubles are equal concerning a delta.
   assertEquals(String, float, float, float)   - 
Static method in class org.testng. AssertJUnit 
 Asserts that two floats are equal concerning a delta.
   assertEquals(float, float, float)   - 
Static method in class org.testng. AssertJUnit 
 Asserts that two floats are equal concerning a delta.
   assertEquals(String, long, long)   - 
Static method in class org.testng. AssertJUnit 
 Asserts that two longs are equal.
   assertEquals(long, long)   - 
Static method in class org.testng. AssertJUnit 
 Asserts that two longs are equal.
   assertEquals(String, boolean, boolean)   - 
Static method in class org.testng. AssertJUnit 
 Asserts that two booleans are equal.
   assertEquals(boolean, boolean)   - 
Static method in class org.testng. AssertJUnit 
 Asserts that two booleans are equal.
   assertEquals(String, byte, byte)   - 
Static method in class org.testng. AssertJUnit 
 Asserts that two bytes are equal.
   assertEquals(byte, byte)   - 
Static method in class org.testng. AssertJUnit 
 Asserts that two bytes are equal.
   assertEquals(String, char, char)   - 
Static method in class org.testng. AssertJUnit 
 Asserts that two chars are equal.
   assertEquals(char, char)   - 
Static method in class org.testng. AssertJUnit 
 Asserts that two chars are equal.
   assertEquals(String, short, short)   - 
Static method in class org.testng. AssertJUnit 
 Asserts that two shorts are equal.
   assertEquals(short, short)   - 
Static method in class org.testng. AssertJUnit 
 Asserts that two shorts are equal.
   assertEquals(String, int, int)   - 
Static method in class org.testng. AssertJUnit 
 Asserts that two ints are equal.
   assertEquals(int, int)   - 
Static method in class org.testng. AssertJUnit 
 Asserts that two ints are equal.
   assertEquals(byte[], byte[])   - 
Static method in class org.testng. AssertJUnit 
  
   assertEquals(String, byte[], byte[])   - 
Static method in class org.testng. AssertJUnit 
  
   assertEqualsNoOrder(Object[], Object[], String)   - 
Static method in class org.testng. Assert 
 Asserts that two arrays contain the same elements in no particular order.
   assertEqualsNoOrder(Object[], Object[])   - 
Static method in class org.testng. Assert 
 Asserts that two arrays contain the same elements in no particular order.
   assertFalse(boolean, String)   - 
Static method in class org.testng. Assert 
 Asserts that a condition is false.
   assertFalse(boolean)   - 
Static method in class org.testng. Assert 
 Asserts that a condition is false.
   assertFalse(String, boolean)   - 
Static method in class org.testng. AssertJUnit 
 Asserts that a condition is false.
   assertFalse(boolean)   - 
Static method in class org.testng. AssertJUnit 
 Asserts that a condition is false.
   assertFile(File, String)   - 
Static method in class org.testng. FileAssert 
 Asserts that a  tstvalue  is a proper directory.
   assertFile(File)   - 
Static method in class org.testng. FileAssert 
  
   AssertJUnit   - Class in  org.testng  A set of assert methods.   AssertJUnit()   - 
Constructor for class org.testng. AssertJUnit 
 Protect constructor since it is a static only class
   assertLength(File, long, String)   - 
Static method in class org.testng. FileAssert 
 Asserts that a  tstvalue  is a file of exactly  expected  characters
 or a directory of exactly  expected  entries.
   assertLength(File, long)   - 
Static method in class org.testng. FileAssert 
  
   assertMaxLength(File, long, String)   - 
Static method in class org.testng. FileAssert 
 Asserts that a  tstvalue  is a file of at most  expected  characters
 or a directory of at most  expected  entries.
   assertMaxLength(File, long)   - 
Static method in class org.testng. FileAssert 
  
   assertMinLength(File, long, String)   - 
Static method in class org.testng. FileAssert 
 Asserts that a  tstvalue  is a file of at least  expected  characters
 or a directory of at least  expected  entries.
   assertMinLength(File, long)   - 
Static method in class org.testng. FileAssert 
  
   assertNotEquals(Object, Object, String)   - 
Static method in class org.testng. Assert 
  
   assertNotEquals(Object, Object)   - 
Static method in class org.testng. Assert 
  
   assertNotEquals(float, float, float, String)   - 
Static method in class org.testng. Assert 
  
   assertNotEquals(float, float, float)   - 
Static method in class org.testng. Assert 
  
   assertNotEquals(double, double, double, String)   - 
Static method in class org.testng. Assert 
  
   assertNotEquals(double, double, double)   - 
Static method in class org.testng. Assert 
  
   assertNotNull(Object)   - 
Static method in class org.testng. Assert 
 Asserts that an object isn't null.
   assertNotNull(Object, String)   - 
Static method in class org.testng. Assert 
 Asserts that an object isn't null.
   assertNotNull(Object)   - 
Static method in class org.testng. AssertJUnit 
 Asserts that an object isn't null.
   assertNotNull(String, Object)   - 
Static method in class org.testng. AssertJUnit 
 Asserts that an object isn't null.
   assertNotSame(Object, Object, String)   - 
Static method in class org.testng. Assert 
 Asserts that two objects do not refer to the same objects.
   assertNotSame(Object, Object)   - 
Static method in class org.testng. Assert 
 Asserts that two objects do not refer to the same object.
   assertNotSame(String, Object, Object)   - 
Static method in class org.testng. AssertJUnit 
 Asserts that two objects refer to the same object.
   assertNotSame(Object, Object)   - 
Static method in class org.testng. AssertJUnit 
 Asserts that two objects refer to the same object.
   assertNull(Object)   - 
Static method in class org.testng. Assert 
 Asserts that an object is null.
   assertNull(Object, String)   - 
Static method in class org.testng. Assert 
 Asserts that an object is null.
   assertNull(Object)   - 
Static method in class org.testng. AssertJUnit 
 Asserts that an object is null.
   assertNull(String, Object)   - 
Static method in class org.testng. AssertJUnit 
 Asserts that an object is null.
   assertReadable(File, String)   - 
Static method in class org.testng. FileAssert 
 Asserts that a  tstvalue  is readable.
   assertReadable(File)   - 
Static method in class org.testng. FileAssert 
  
   assertReadWrite(File, String)   - 
Static method in class org.testng. FileAssert 
 Asserts that a  tstvalue  is readable and writeable.
   assertReadWrite(File)   - 
Static method in class org.testng. FileAssert 
  
   assertSame(Object, Object, String)   - 
Static method in class org.testng. Assert 
 Asserts that two objects refer to the same object.
   assertSame(Object, Object)   - 
Static method in class org.testng. Assert 
 Asserts that two objects refer to the same object.
   assertSame(String, Object, Object)   - 
Static method in class org.testng. AssertJUnit 
 Asserts that two objects refer to the same object.
   assertSame(Object, Object)   - 
Static method in class org.testng. AssertJUnit 
 Asserts that two objects refer to the same object.
   assertTrue(boolean, String)   - 
Static method in class org.testng. Assert 
 Asserts that a condition is true.
   assertTrue(boolean)   - 
Static method in class org.testng. Assert 
 Asserts that a condition is true.
   assertTrue(String, boolean)   - 
Static method in class org.testng. AssertJUnit 
 Asserts that a condition is true.
   assertTrue(boolean)   - 
Static method in class org.testng. AssertJUnit 
 Asserts that a condition is true.
   assertWriteable(File, String)   - 
Static method in class org.testng. FileAssert 
 Asserts that a  tstvalue  is writeable.
   assertWriteable(File)   - 
Static method in class org.testng. FileAssert 
  
 
 
    
 B  
 
   BeforeClass   - Annotation Type in  org.testng.annotations      beforeConfiguration(ITestResult)   - 
Method in interface org.testng. IConfigurationListener2 
 Invoked before a configuration method is invoked.
   beforeConfiguration(ITestResult)   - 
Method in class org.testng. TestListenerAdapter 
  
   beforeConfiguration(ITestResult)   - 
Method in class org.testng. TestNG.ExitCodeListener 
  
   BeforeGroups   - Annotation Type in  org.testng.annotations      beforeInvocation(IInvokedMethod, ITestResult)   - 
Method in interface org.testng. IInvokedMethodListener 
  
   beforeInvocation(IInvokedMethod, ITestResult, ITestContext)   - 
Method in interface org.testng. IInvokedMethodListener2 
 To be implemented if the method needs a handle to contextual information.
   beforeInvocation(IInvokedMethod, ITestResult)   - 
Method in class org.testng. SuiteRunner 
  
   BeforeMethod   - Annotation Type in  org.testng.annotations      BeforeSuite   - Annotation Type in  org.testng.annotations      BeforeTest   - Annotation Type in  org.testng.annotations    
 
    
 C  
 
   canRunFromClass(IClass)   - 
Method in interface org.testng. ITestNGMethod 
 Returns if this ITestNGMethod can be invoked from within IClass.
   ClassMethodMap   - Class in  org.testng  This class maintains a map of   > .   ClassMethodMap(List<ITestNGMethod>, XmlMethodSelector)   - 
Constructor for class org.testng. ClassMethodMap 
  
   clear()   - 
Method in class org.testng. ClassMethodMap 
  
   clear()   - 
Static method in class org.testng. Reporter 
 Erase the content of all the output generated so far.
   clone()   - 
Method in interface org.testng. ITestNGMethod 
  
   clone()   - 
Method in class org.testng.xml. XmlClass 
 Clone an XmlClass by copying all its components.
   clone()   - 
Method in class org.testng.xml. XmlSuite 
 
 Note that this is not a full clone:  XmlTest children are not cloned by this
 method.
   clone()   - 
Method in class org.testng.xml. XmlTest 
 Clone the  source   XmlTest  by including:
 - test attributes
 - groups definitions
 - parameters

 The <classes> sub element is ignored for the moment.
   CommandLineArgs   - Class in  org.testng      CommandLineArgs()   - 
Constructor for class org.testng. CommandLineArgs 
  
   commandLineMethods   - 
Variable in class org.testng. CommandLineArgs 
  
   compareTo(IWorker<ISuite>)   - 
Method in class org.testng. SuiteRunnerWorker 
  
   CONFIG_FAILURE_POLICY   - 
Static variable in class org.testng. CommandLineArgs 
  
   configFailurePolicy   - 
Variable in class org.testng. CommandLineArgs 
  
   Configuration   - Annotation Type in  org.testng.annotations   Deprecated.   Use @BeforeSuite, @AfterSuite, @BeforeTest, @AfterTest,
 \@BeforeGroups, @AfterGroups, @BeforeClass, @AfterClass, @BeforeMethod,
 \@AfterMethod    configure(CommandLineArgs)   - 
Method in class org.testng. TestNG 
 Configure the TestNG instance based on the command line parameters.
   configure(Map)   - 
Method in class org.testng. TestNG 
  Deprecated.   use new configure(CommandLineArgs) method 
   CONTINUE   - 
Static variable in class org.testng.xml. XmlSuite 
  
   ConversionUtils   - Class in  org.testng  Helper methods used by the Eclipse plug-in when converting tests from JUnit.   ConversionUtils()   - 
Constructor for class org.testng. ConversionUtils 
  
   Converter   - Class in  org.testng  Convert XML files to YAML and vice versa.   Converter()   - 
Constructor for class org.testng. Converter 
  
   createBootclasspath()   - 
Method in class org.testng. TestNGAntTask 
 Adds a path to the bootclasspath.
   createClasspath()   - 
Method in class org.testng. TestNGAntTask 
 Adds path to classpath used for tests.
   createITestNGMethod(ITestNGMethod, Method)   - 
Static method in class org.testng. TestNGUtils 
 Create an ITestNGMethod for @code{method} based on @code{existingMethod}, which needs
 to belong to the same class.
   createJvmarg()   - 
Method in class org.testng. TestNGAntTask 
  
   createListener(Class<? extends ITestNGListener>)   - 
Method in interface org.testng. ITestNGListenerFactory 
 Create and return an instance of the listener class passed in parameter.
   createModule(ITestContext, Class<?>)   - 
Method in interface org.testng. IModuleFactory 
  
   createWatchdog()   - 
Method in class org.testng. TestNGAntTask 
  
   createWorkers(List<ITestNGMethod>)   - 
Method in class org.testng. TestRunner 
 Create a list of workers to run the methods passed in parameter.
 
 
    
 D  
 
   DATA_PROVIDER_THREAD_COUNT   - 
Static variable in class org.testng. CommandLineArgs 
  
   DataProvider   - Annotation Type in  org.testng.annotations  Mark a method as supplying data for a test method.   dataProviderThreadCount   - 
Variable in class org.testng. CommandLineArgs 
  
   DEBUG   - 
Static variable in class org.testng. CommandLineArgs 
  
   debug   - 
Variable in class org.testng. CommandLineArgs 
  
   DEFAULT_ALLOW_RETURN_VALUES   - 
Static variable in class org.testng.xml. XmlSuite 
  
   DEFAULT_COMMAND_LINE_SUITE_NAME   - 
Static variable in class org.testng. TestNG 
 The default name for a suite launched from the command line
   DEFAULT_COMMAND_LINE_TEST_NAME   - 
Static variable in class org.testng. TestNG 
 The default name for a test launched from the command line
   DEFAULT_CONFIG_FAILURE_POLICY   - 
Static variable in class org.testng.xml. XmlSuite 
 Whether to SKIP or CONTINUE to re-attempt failed configuration methods.
   DEFAULT_DATA_PROVIDER_THREAD_COUNT   - 
Static variable in class org.testng.xml. XmlSuite 
 Thread count for the data provider pool
   DEFAULT_GROUP_BY_INSTANCES   - 
Static variable in class org.testng.xml. XmlSuite 
 By default, a method failing will cause all instances of that class to skip
   DEFAULT_JUNIT   - 
Static variable in class org.testng.xml. XmlSuite 
 JUnit compatibility flag.
   DEFAULT_MIXED   - 
Static variable in class org.testng.xml. XmlSuite 
 mixed mode flag.
   DEFAULT_OUTPUTDIR   - 
Static variable in class org.testng. TestNG 
 The default name of the result's output directory (keep public, used by Eclipse).
   DEFAULT_PARALLEL   - 
Static variable in class org.testng.xml. XmlSuite 
  
   DEFAULT_PRESERVE_ORDER   - 
Static variable in class org.testng.xml. XmlSuite 
  
   DEFAULT_SKIP_FAILED_INVOCATION_COUNTS   - 
Static variable in class org.testng.xml. XmlSuite 
  
   DEFAULT_THREAD_COUNT   - 
Static variable in class org.testng.xml. XmlSuite 
 The thread count.
   DEFAULT_TIMEOUT_MS   - 
Static variable in class org.testng.xml. XmlTest 
  
   DEFAULT_VERBOSE   - 
Static variable in class org.testng. TestNG 
  
   DEFAULT_VERBOSE   - 
Static variable in class org.testng.xml. XmlSuite 
 The suite verbose flag.
   DependencyMap   - Class in  org.testng  Helper class to keep track of dependencies.   DependencyMap(ITestNGMethod[])   - 
Constructor for class org.testng. DependencyMap 
  
   deserialize(String)   - 
Static method in class org.testng. ReporterConfig 
  
 
 
    
 E  
 
   equals(Object)   - 
Method in class org.testng.xml. XmlClass 
  
   equals(Object)   - 
Method in class org.testng.xml. XmlInclude 
  
   equals(Object)   - 
Method in class org.testng.xml. XmlMethodSelector 
  
   equals(Object)   - 
Method in class org.testng.xml. XmlPackage 
  
   equals(Object)   - 
Method in class org.testng.xml. XmlSuite 
  
   equals(Object)   - 
Method in class org.testng.xml. XmlTest 
  
   EXCLUDED_GROUPS   - 
Static variable in class org.testng. CommandLineArgs 
  
   excludedGroups   - 
Variable in class org.testng. CommandLineArgs 
  
   execute()   - 
Method in class org.testng. TestNGAntTask 
 Launches TestNG in a new JVM.
   executeAsForked(CommandlineJava, ExecuteWatchdog)   - 
Method in class org.testng. TestNGAntTask 
 Executes the command line as a new process.
   ExpectedExceptions   - Annotation Type in  org.testng.annotations   Deprecated.   Use @Test(expectedExceptions = "...")  
 
    
 F  
 
   Factory   - Annotation Type in  org.testng.annotations  Marks a method as a factory that returns objects that will be used by TestNG
 as Test classes.   fail(String, Throwable)   - 
Static method in class org.testng. Assert 
 Fails a test with the given message and wrapping the original exception.
   fail(String)   - 
Static method in class org.testng. Assert 
 Fails a test with the given message.
   fail()   - 
Static method in class org.testng. Assert 
 Fails a test with no message.
   fail(String)   - 
Static method in class org.testng. AssertJUnit 
 Fails a test with the given message.
   fail()   - 
Static method in class org.testng. AssertJUnit 
 Fails a test with no message.
   fail(String, Throwable)   - 
Static method in class org.testng. FileAssert 
 Fails a test with the given message and wrapping the original exception.
   fail(String)   - 
Static method in class org.testng. FileAssert 
 Fails a test with the given message.
   fail()   - 
Static method in class org.testng. FileAssert 
 Fails a test with no message.
   failed()   - 
Method in class org.testng. SuiteRunState 
  
   FAILURE   - 
Static variable in interface org.testng. ITestResult 
  
   FileAssert   - Class in  org.testng  Assertion tool for File centric assertions.   findMethodParameters(XmlTest)   - 
Method in interface org.testng. ITestNGMethod 
  
   findTestClasses()   - 
Method in interface org.testng. ITestClassFinder 
  
 
 
    
 G  
 
   generateReport(List<XmlSuite>, List<ISuite>, String)   - 
Method in interface org.testng. IReporter 
 Generate a report for the given suites into the specified output directory.
   getAfterClassMethods()   - 
Method in interface org.testng. ITestClass 
 Returns all the methods that should be invoked
 after all the tests have been run on this class.
   getAfterClassMethods(Class)   - 
Method in interface org.testng. ITestMethodFinder 
  
   getAfterGroups()   - 
Method in interface org.testng.annotations. IConfigurationAnnotation 
 The list of groups that this configuration method will run after.
   getAfterGroups()   - 
Method in interface org.testng. ITestNGMethod 
  
   getAfterGroupsConfigurationMethods(Class)   - 
Method in interface org.testng. ITestMethodFinder 
  
   getAfterGroupsMethods()   - 
Method in interface org.testng. ITestClass 
 Returns all @Configuration methods that should be invoked after certain groups.
   getAfterSuite()   - 
Method in interface org.testng.annotations. IConfigurationAnnotation 
 If true, the annotated method will be run after all tests in this suite
 have run.
   getAfterSuiteMethods()   - 
Method in interface org.testng. ITestClass 
 Returns all the methods that should be invoked
 after the suite has run.
   getAfterSuiteMethods(Class)   - 
Method in interface org.testng. ITestMethodFinder 
  
   getAfterSuiteMethods()   - 
Method in class org.testng. TestRunner 
  
   getAfterTest()   - 
Method in interface org.testng.annotations. IConfigurationAnnotation 
 If true, the annotated method will be run after all every test.
   getAfterTestClass()   - 
Method in interface org.testng.annotations. IConfigurationAnnotation 
 If true, the annotated method will be run after all the tests in the test
 class have been run.
   getAfterTestConfigurationMethods()   - 
Method in interface org.testng. ITestClass 
 Returns all @Configuration methods that should be invoked last before any others
 in the current test.
   getAfterTestConfigurationMethods(Class)   - 
Method in interface org.testng. ITestMethodFinder 
  
   getAfterTestConfigurationMethods()   - 
Method in class org.testng. TestRunner 
  
   getAfterTestMethod()   - 
Method in interface org.testng.annotations. IConfigurationAnnotation 
 If true, the annotated method will be run after any test method is invoked.
   getAfterTestMethods()   - 
Method in interface org.testng. ITestClass 
 Returns all the methods that should be invoked
 after a test method completes.
   getAfterTestMethods(Class)   - 
Method in interface org.testng. ITestMethodFinder 
  
   getAllInvokedMethods()   - 
Method in interface org.testng. ISuite 
  
   getAllInvokedMethods()   - 
Method in class org.testng. SuiteRunner 
  
   getAllMethods()   - 
Method in interface org.testng. IResultMap 
  
   getAllMethods()   - 
Method in interface org.testng. ISuite 
  
   getAllMethods()   - 
Method in class org.testng. SuiteRunner 
  
   getAllowReturnValues()   - 
Method in class org.testng.xml. XmlSuite 
  
   getAllowReturnValues()   - 
Method in class org.testng.xml. XmlTest 
  
   getAllParameters()   - 
Method in class org.testng.xml. XmlSuite 
  
   getAllResults()   - 
Method in interface org.testng. IResultMap 
  
   getAllTestMethods()   - 
Method in interface org.testng. ITestContext 
  
   getAllTestMethods()   - 
Method in class org.testng. TestListenerAdapter 
  
   getAllTestMethods()   - 
Method in class org.testng. TestRunner 
  
   getAlwaysRun()   - 
Method in interface org.testng.annotations. IConfigurationAnnotation 
 Used only for after type of configuration methods.
   getAlwaysRun()   - 
Method in interface org.testng.annotations. ITestAnnotation 
 If set to true, this test method will always be run even if it depends
 on a method that failed.
   getAnnotationFinder()   - 
Method in interface org.testng. ISuite 
  
   getAnnotationFinder()   - 
Method in class org.testng. SuiteRunner 
 Returns the annotation finder for the given annotation type.
   getAnnotationTransformer()   - 
Method in class org.testng. TestNG 
  
   getAttribute(String)   - 
Method in interface org.testng. IAttributes 
  
   getAttribute(String)   - 
Method in class org.testng. SuiteRunner 
  
   getAttribute(String)   - 
Method in class org.testng. TestRunner 
  
   getAttributeNames()   - 
Method in interface org.testng. IAttributes 
  
   getAttributeNames()   - 
Method in class org.testng. SuiteRunner 
  
   getAttributeNames()   - 
Method in class org.testng. TestRunner 
  
   getBeforeClassMethods()   - 
Method in interface org.testng. ITestClass 
 Return all the methods that should be invoked
 after the test class has been created and before
 any of its test methods is invoked.
   getBeforeClassMethods(Class)   - 
Method in interface org.testng. ITestMethodFinder 
  
   getBeforeGroups()   - 
Method in interface org.testng.annotations. IConfigurationAnnotation 
 The list of groups that this configuration method will run before.
   getBeforeGroups()   - 
Method in interface org.testng. ITestNGMethod 
 Before and After groups
   getBeforeGroupsConfigurationMethods(Class)   - 
Method in interface org.testng. ITestMethodFinder 
  
   getBeforeGroupsMethods()   - 
Method in interface org.testng. ITestClass 
 Returns all @Configuration methods that should be invoked before certain groups.
   getBeforeSuite()   - 
Method in interface org.testng.annotations. IConfigurationAnnotation 
 If true, the annotated method will be run before this suite starts.
   getBeforeSuiteMethods()   - 
Method in interface org.testng. ITestClass 
 Returns All the methods that should be invoked
 before the suite is run.
   getBeforeSuiteMethods(Class)   - 
Method in interface org.testng. ITestMethodFinder 
  
   getBeforeSuiteMethods()   - 
Method in class org.testng. TestRunner 
  
   getBeforeTest()   - 
Method in interface org.testng.annotations. IConfigurationAnnotation 
 If true, the annotated method will be run before every test
   getBeforeTestClass()   - 
Method in interface org.testng.annotations. IConfigurationAnnotation 
 If true, the annotated method will be run after the test class is instantiated
 and before the test method is invoked.
   getBeforeTestConfigurationMethods()   - 
Method in interface org.testng. ITestClass 
 Returns all @Configuration methods that should be invoked before any others in the
 current test.
   getBeforeTestConfigurationMethods(Class)   - 
Method in interface org.testng. ITestMethodFinder 
  
   getBeforeTestConfigurationMethods()   - 
Method in class org.testng. TestRunner 
  
   getBeforeTestMethod()   - 
Method in interface org.testng.annotations. IConfigurationAnnotation 
 If true, the annotated method will be run before any test method is invoked.
   getBeforeTestMethods()   - 
Method in interface org.testng. ITestClass 
 Returns all the methods that should be invoked
 before a test method is invoked.
   getBeforeTestMethods(Class)   - 
Method in interface org.testng. ITestMethodFinder 
  
   getChildSuites()   - 
Method in class org.testng.xml. XmlSuite 
  
   getClasses()   - 
Method in class org.testng.xml. XmlTest 
  
   getClassName()   - 
Method in class org.testng. ReporterConfig 
  
   getClassName()   - 
Method in class org.testng.xml. XmlMethodSelector 
  
   getConfigFailurePolicy()   - 
Method in class org.testng. TestNG 
 Returns the configuration failure policy.
   getConfigFailurePolicy()   - 
Method in class org.testng.xml. XmlSuite 
 Returns the configuration failure policy.
   getConfiguration()   - 
Method in class org.testng. TestNG 
  
   getConfigurationFailures()   - 
Method in class org.testng. TestListenerAdapter 
  
   getConfigurationListeners()   - 
Method in class org.testng. TestRunner 
  
   getConfigurationSkips()   - 
Method in class org.testng. TestListenerAdapter 
  
   getConstructorOrMethod()   - 
Method in interface org.testng. ITestNGMethod 
  
   getCurrentInvocationCount()   - 
Method in interface org.testng. ITestNGMethod 
  
   getCurrentTestResult()   - 
Static method in class org.testng. Reporter 
  
   getCurrentXmlTest()   - 
Method in interface org.testng. ITestContext 
  
   getCurrentXmlTest()   - 
Method in class org.testng. TestRunner 
  
   getDataProvider()   - 
Method in interface org.testng.annotations. ITestAnnotation 
  
   getDataProviderClass()   - 
Method in interface org.testng.annotations. ITestAnnotation 
  
   getDataProviderThreadCount()   - 
Method in class org.testng.xml. XmlSuite 
  
   getDate()   - 
Method in interface org.testng. IInvokedMethod 
  
   getDate()   - 
Method in interface org.testng. ITestNGMethod 
  
   getDefault()   - 
Static method in class org.testng. TestNG 
  Deprecated.   since 5.1 
   getDefaultSuiteName()   - 
Method in class org.testng. TestNG 
  
   getDefaultTestName()   - 
Method in class org.testng. TestNG 
  
   getDependsOnGroups()   - 
Method in interface org.testng.annotations. ITestOrConfiguration 
 The list of groups this method depends on.
   getDependsOnMethods()   - 
Method in interface org.testng.annotations. ITestOrConfiguration 
 The list of methods this method depends on.
   getDescription()   - 
Method in interface org.testng.annotations. ITestOrConfiguration 
 The description for this method, which will be shown in the reports.
   getDescription()   - 
Method in interface org.testng. ITestNGMethod 
  
   getDescription()   - 
Method in class org.testng.xml. XmlInclude 
  
   getEnabled()   - 
Method in interface org.testng.annotations. IParameterizable 
 Whether this annotation is enabled.
   getEnabled()   - 
Method in interface org.testng. ITestNGMethod 
  
   getEnd()   - 
Method in class org.testng. TestNG 
  
   getEndDate()   - 
Method in interface org.testng. ITestContext 
 When this test stopped running.
   getEndDate()   - 
Method in class org.testng. TestRunner 
  
   getEndMillis()   - 
Method in interface org.testng. ITestResult 
  
   getExclude()   - 
Method in class org.testng.xml. XmlPackage 
  
   getExcludedGroups()   - 
Method in interface org.testng. ITestContext 
  
   getExcludedGroups()   - 
Method in class org.testng. TestRunner 
  
   getExcludedGroups()   - 
Method in class org.testng.xml. XmlSuite 
  
   getExcludedGroups()   - 
Method in class org.testng.xml. XmlTest 
  
   getExcludedMethods()   - 
Method in interface org.testng. ISuite 
  
   getExcludedMethods()   - 
Method in interface org.testng. ITestContext 
  
   getExcludedMethods()   - 
Method in class org.testng. SuiteRunner 
  
   getExcludedMethods()   - 
Method in class org.testng. TestRunner 
  
   getExcludedMethods()   - 
Method in class org.testng.xml. XmlClass 
  
   getExpectedExceptions()   - 
Method in interface org.testng.annotations. ITestAnnotation 
  
   getExpectedExceptionsMessageRegExp()   - 
Method in interface org.testng.annotations. ITestAnnotation 
  
   getExpression()   - 
Method in class org.testng.xml. XmlMethodSelector 
  
   getExpression()   - 
Method in class org.testng.xml. XmlTest 
  
   getFailedButWithinSuccessPercentageTests()   - 
Method in interface org.testng. ITestContext 
  
   getFailedButWithinSuccessPercentageTests()   - 
Method in class org.testng. TestListenerAdapter 
  
   getFailedButWithinSuccessPercentageTests()   - 
Method in class org.testng. TestRunner 
  
   getFailedConfigurations()   - 
Method in interface org.testng. ITestContext 
 Retrieves information about the failed configuration method invocations.
   getFailedConfigurations()   - 
Method in class org.testng. TestRunner 
  
   getFailedInvocationNumbers()   - 
Method in interface org.testng. ITestNGMethod 
  
   getFailedTests()   - 
Method in interface org.testng. ITestContext 
  
   getFailedTests()   - 
Method in class org.testng. TestListenerAdapter 
  
   getFailedTests()   - 
Method in class org.testng. TestRunner 
  
   getFailedTests(ITestNGMethod)   - 
Method in class org.testng. TestRunner 
  
   getFileName()   - 
Method in class org.testng.xml. XmlSuite 
  
   getGroupByInstances()   - 
Method in class org.testng.xml. XmlSuite 
  
   getGroupByInstances()   - 
Method in class org.testng.xml. XmlTest 
  
   getGroups()   - 
Method in interface org.testng.annotations. ITestOrConfiguration 
 The list of groups this class/method belongs to.
   getGroups()   - 
Method in interface org.testng. ITestNGMethod 
  
   getGroupsDependedUpon()   - 
Method in interface org.testng. ITestNGMethod 
  
   getGuiceModules(Class<? extends Module>)   - 
Method in interface org.testng. ITestContext 
  
   getGuiceModules(Class<? extends Module>)   - 
Method in class org.testng. TestRunner 
  
   getHost()   - 
Method in interface org.testng. ISuite 
  
   getHost()   - 
Method in interface org.testng. ITestContext 
  
   getHost()   - 
Method in interface org.testng. ITestResult 
  
   getHost()   - 
Method in class org.testng. SuiteRunner 
  
   getHost()   - 
Method in class org.testng. TestRunner 
  
   getIClass(Class)   - 
Method in interface org.testng. ITestClassFinder 
 Return the IClass for a given class
   getId()   - 
Method in interface org.testng. ITestNGMethod 
  
   getInclude()   - 
Method in class org.testng.xml. XmlPackage 
  
   getIncludedGroups()   - 
Method in interface org.testng. ITestContext 
  
   getIncludedGroups()   - 
Method in class org.testng. TestRunner 
  
   getIncludedGroups()   - 
Method in class org.testng.xml. XmlSuite 
  
   getIncludedGroups()   - 
Method in class org.testng.xml. XmlTest 
  
   getIncludedMethods()   - 
Method in class org.testng.xml. XmlClass 
  
   getIndex()   - 
Method in class org.testng.xml. XmlClass 
 Note that this attribute does not come from the XML file, it's calculated
 internally and represents the order in which this class was found in its
 <test> tag.
   getIndex()   - 
Method in class org.testng.xml. XmlInclude 
  
   getIndex()   - 
Method in class org.testng.xml. XmlTest 
 Note that this attribute does not come from the XML file, it's calculated
 internally and represents the order in which this test tag was found in its
 <suite> tag.
   getInheritGroups()   - 
Method in interface org.testng.annotations. IConfigurationAnnotation 
 If true, this @Configuration method will belong to groups specified in the
 \@Test annotation on the class (if any).
   getInjector(List<Module>)   - 
Method in interface org.testng. ITestContext 
  
   getInjector(List<Module>)   - 
Method in class org.testng. TestRunner 
  
   getInstance()   - 
Method in interface org.testng. IInstanceInfo 
  
   getInstance()   - 
Method in interface org.testng. IMethodInstance 
  
   getInstance()   - 
Method in interface org.testng. ITestNGMethod 
  
   getInstance()   - 
Method in interface org.testng. ITestResult 
 The instance on which this method was run.
   getInstanceClass()   - 
Method in interface org.testng. IInstanceInfo 
  
   getInstanceCount()   - 
Method in interface org.testng. IClass 
  
   getInstanceCount()   - 
Method in interface org.testng. ITestClass 
  
   getInstanceHashCodes()   - 
Method in interface org.testng. IClass 
  
   getInstanceHashCodes()   - 
Method in interface org.testng. ITestClass 
 TODO cquezel JavaDoc.
   getInstanceHashCodes()   - 
Method in interface org.testng. ITestNGMethod 
 Needed for serialization.
   getInstanceName()   - 
Method in interface org.testng. ITestResult 
  
   getInstances(boolean)   - 
Method in interface org.testng. IClass 
  
   getInstances()   - 
Method in interface org.testng. IMethodInstance 
  Deprecated.   Use getInstance() 
   getInstances(boolean)   - 
Method in interface org.testng. ITestClass 
 Returns all the instances the methods will be invoked upon.
   getInstances()   - 
Method in interface org.testng. ITestNGMethod 
  Deprecated.   Use getInstance(). 
   getInvocationCount()   - 
Method in interface org.testng.annotations. ITestAnnotation 
 Returns the number of times this method should be invoked.
   getInvocationCount()   - 
Method in interface org.testng. ITestNGMethod 
  
   getInvocationNumbers()   - 
Method in interface org.testng. ITestNGMethod 
 Which invocation numbers of this method should be used (only applicable
 if it uses a data provider).
   getInvocationNumbers()   - 
Method in class org.testng.xml. XmlInclude 
  
   getInvocationNumbers(String)   - 
Method in class org.testng.xml. XmlTest 
 Convenience method to cache the ordering numbers for methods.
   getInvocationTimeOut()   - 
Method in interface org.testng. ITestNGMethod 
 The time under which all invocationCount methods need to complete by.
   getInvokedAfterClassMethods()   - 
Method in class org.testng. ClassMethodMap 
  
   getInvokedBeforeClassMethods()   - 
Method in class org.testng. ClassMethodMap 
  
   getInvokedMethods()   - 
Method in interface org.testng. ISuite 
  Deprecated.   Use getAllInvokedMthods(). 
   getInvokedMethods()   - 
Method in class org.testng. SuiteRunner 
  
   getInvokedMethods()   - 
Method in class org.testng. TestRunner 
  
   getInvoker()   - 
Method in class org.testng. TestRunner 
  
   getJavaCommand()   - 
Method in class org.testng. TestNGAntTask 
 Creates or returns the already created  CommandlineJava .
   getLanguage()   - 
Method in class org.testng.xml. XmlMethodSelector 
  
   getListeners()   - 
Method in class org.testng.xml. XmlSuite 
  
   getMessage()   - 
Method in exception org.testng. TimeBombSkipException 
  
   getMetaGroups()   - 
Method in class org.testng.xml. XmlTest 
  
   getMethod()   - 
Method in interface org.testng. IMethodInstance 
  
   getMethod()   - 
Method in interface org.testng. ITestNGMethod 
  Deprecated.   This method is deprecated and can now return null. Use
 getConstructorOrMethod() instead. 
   getMethod()   - 
Method in interface org.testng. ITestResult 
  
   getMethodDependingOn(String, ITestNGMethod)   - 
Method in class org.testng. DependencyMap 
  
   getMethodName()   - 
Method in interface org.testng. ITestNGMethod 
 Returns the method name.
   getMethodsByGroups()   - 
Method in interface org.testng. ISuite 
 Retrieves the map of groups and their associated test methods.
   getMethodsByGroups()   - 
Method in class org.testng. SuiteRunner 
  
   getMethodsDependedUpon()   - 
Method in interface org.testng. ITestNGMethod 
  
   getMethodSelectors()   - 
Method in class org.testng.xml. XmlSuite 
 Returns the method selectors.
   getMethodSelectors()   - 
Method in class org.testng.xml. XmlTest 
  
   getMethodsThatBelongTo(String, ITestNGMethod)   - 
Method in class org.testng. DependencyMap 
  
   getMissingGroup()   - 
Method in interface org.testng. ITestNGMethod 
 If a group was not found.
   getName()   - 
Method in interface org.testng.annotations. IDataProviderAnnotation 
 The name of this DataProvider.
   getName()   - 
Method in interface org.testng. IClass 
  
   getName()   - 
Method in interface org.testng. ISuite 
  
   getName()   - 
Method in interface org.testng. ITestContext 
 The name of this test.
   getName()   - 
Method in interface org.testng. ITestResult 
  
   getName()   - 
Method in class org.testng. ReporterConfig.Property 
  
   getName()   - 
Method in class org.testng. SuiteRunner 
  
   getName()   - 
Method in class org.testng. TestRunner 
  
   getName()   - 
Method in class org.testng.xml. XmlClass 
  
   getName()   - 
Method in class org.testng.xml. XmlInclude 
  
   getName()   - 
Method in class org.testng.xml. XmlPackage 
  
   getName()   - 
Method in class org.testng.xml. XmlSuite 
 Returns the name.
   getName()   - 
Method in class org.testng.xml. XmlTest 
  
   getObjectFactory()   - 
Method in interface org.testng. ISuite 
  
   getObjectFactory()   - 
Method in class org.testng. SuiteRunner 
  
   getObjectFactory()   - 
Method in class org.testng.xml. XmlSuite 
  
   getObjectFactory2()   - 
Method in interface org.testng. ISuite 
  
   getObjectFactory2()   - 
Method in class org.testng. SuiteRunner 
  
   getOutput()   - 
Static method in class org.testng. Reporter 
  
   getOutput(ITestResult)   - 
Static method in class org.testng. Reporter 
  
   getOutputDirectory()   - 
Method in interface org.testng. ISuite 
  
   getOutputDirectory()   - 
Method in interface org.testng. ITestContext 
  
   getOutputDirectory()   - 
Method in class org.testng. SuiteRunner 
  
   getOutputDirectory()   - 
Method in class org.testng. TestNG 
  
   getOutputDirectory()   - 
Method in class org.testng. TestRunner 
  
   getPackages()   - 
Method in class org.testng.xml. XmlSuite 
  
   getPackages()   - 
Method in class org.testng.xml. XmlTest 
  
   getParallel()   - 
Method in interface org.testng. ISuite 
  
   getParallel()   - 
Method in class org.testng. SuiteRunner 
  
   getParallel()   - 
Method in class org.testng.xml. XmlSuite 
 Returns the parallel mode.
   getParallel()   - 
Method in class org.testng.xml. XmlTest 
  
   getParameter(String)   - 
Method in interface org.testng. ISuite 
  
   getParameter(String)   - 
Method in class org.testng. SuiteRunner 
 FIXME: should be removed?
   getParameter(String)   - 
Method in class org.testng.xml. XmlSuite 
 Returns the parameter defined in this suite only.
   getParameter(String)   - 
Method in class org.testng.xml. XmlTest 
  
   getParameterInvocationCount()   - 
Method in interface org.testng. ITestNGMethod 
  
   getParameterOutput()   - 
Method in interface org.testng. IExtraOutput 
  
   getParameters()   - 
Method in interface org.testng.annotations. IParameterizable 
  Deprecated.   Use @Parameters 
   getParameters()   - 
Method in interface org.testng. IConfigureCallBack 
  
   getParameters()   - 
Method in interface org.testng. IHookCallBack 
  
   getParameters()   - 
Method in interface org.testng. ITestResult 
  
   getParameters()   - 
Method in class org.testng.xml. XmlInclude 
  
   getParameters()   - 
Method in class org.testng.xml. XmlSuite 
 Gets the parameters that apply to tests in this suite. 
 Set of parameters for a suite is appended with parameters from parent suite.
   getParameters()   - 
Method in class org.testng.xml. XmlTest 
 Returns a merge of the the test parameters and its parent suite parameters.
   getParentSuite()   - 
Method in class org.testng.xml. XmlSuite 
  
   getPassedConfigurations()   - 
Method in interface org.testng. ITestContext 
 Retrieves information about the successful configuration method invocations.
   getPassedConfigurations()   - 
Method in class org.testng. TestRunner 
  
   getPassedTests()   - 
Method in interface org.testng. ITestContext 
  
   getPassedTests()   - 
Method in class org.testng. TestListenerAdapter 
  
   getPassedTests()   - 
Method in class org.testng. TestRunner 
  
   getPassedTests(ITestNGMethod)   - 
Method in class org.testng. TestRunner 
  
   getPreserveOrder()   - 
Method in class org.testng.xml. XmlSuite 
  
   getPreserveOrder()   - 
Method in class org.testng.xml. XmlTest 
  
   getPriority()   - 
Method in interface org.testng.annotations. ITestAnnotation 
 The scheduling priority.
   getPriority()   - 
Method in interface org.testng. ITestNGMethod 
 The scheduling priority.
   getPriority()   - 
Method in class org.testng. SuiteRunnerWorker 
  
   getPriority()   - 
Method in class org.testng.xml. XmlMethodSelector 
  
   getProperties()   - 
Method in class org.testng. ReporterConfig 
  
   getPropertyFileName()   - 
Method in interface org.testng. ISuiteResult 
  
   getRealClass()   - 
Method in interface org.testng. IClass 
  
   getRealClass()   - 
Method in interface org.testng. ITestNGMethod 
  
   getReporters()   - 
Method in class org.testng. SuiteRunner 
  
   getReporters()   - 
Method in class org.testng. TestNG 
  
   getResults(ITestNGMethod)   - 
Method in interface org.testng. IResultMap 
  
   getResults()   - 
Method in interface org.testng. ISuite 
  
   getResults()   - 
Method in class org.testng. SuiteRunner 
  
   getRetryAnalyzer()   - 
Method in interface org.testng.annotations. ITestAnnotation 
  
   getRetryAnalyzer()   - 
Method in interface org.testng. ITestNGMethod 
  
   getSequential()   - 
Method in interface org.testng.annotations. ITestAnnotation 
  
   getServiceLoaderListeners()   - 
Method in class org.testng. TestNG 
  
   getSingleThreaded()   - 
Method in interface org.testng.annotations. ITestAnnotation 
  
   getSkippedConfigurations()   - 
Method in interface org.testng. ITestContext 
 Retrieves information about the skipped configuration method invocations.
   getSkippedConfigurations()   - 
Method in class org.testng. TestRunner 
  
   getSkippedTests()   - 
Method in interface org.testng. ITestContext 
  
   getSkippedTests()   - 
Method in class org.testng. TestListenerAdapter 
  
   getSkippedTests()   - 
Method in class org.testng. TestRunner 
  
   getSkippedTests(ITestNGMethod)   - 
Method in class org.testng. TestRunner 
  
   getStart()   - 
Method in class org.testng. TestNG 
  
   getStartDate()   - 
Method in interface org.testng. ITestContext 
 When this test started running.
   getStartDate()   - 
Method in class org.testng. TestRunner 
  
   getStartMillis()   - 
Method in interface org.testng. ITestResult 
  
   getStatus()   - 
Method in interface org.testng. ITestResult 
  
   getStatus()   - 
Method in class org.testng. TestNG 
  
   getSuccessPercentage()   - 
Method in interface org.testng.annotations. ITestAnnotation 
 The percentage of success expected from this method.
   getSuccessPercentage()   - 
Method in interface org.testng. ITestNGMethod 
  
   getSuite()   - 
Method in interface org.testng. ITestContext 
  
   getSuite()   - 
Method in class org.testng. TestRunner 
  
   getSuite()   - 
Method in class org.testng.xml. XmlTest 
 Returns the suite this test is part of.
   getSuiteFileNames()   - 
Method in class org.testng. TestNGAntTask 
  
   getSuiteFiles()   - 
Method in class org.testng.xml. XmlSuite 
 Returns the suite files.
   getSuiteListeners()   - 
Method in class org.testng. TestNG 
  
   getSuiteName()   - 
Method in interface org.testng.annotations. ITestAnnotation 
  
   getSuiteState()   - 
Method in interface org.testng. ISuite 
 Retrieves the shared state for a suite.
   getSuiteState()   - 
Method in class org.testng. SuiteRunner 
  
   getSuiteThreadPoolSize()   - 
Method in class org.testng. TestNG 
  
   getSupportClass()   - 
Method in class org.testng.xml. XmlClass 
  
   getTasks()   - 
Method in class org.testng. SuiteRunnerWorker 
  
   getTest()   - 
Method in class org.testng. TestRunner 
  
   getTest()   - 
Method in class org.testng.xml. XmlSuite 
 Returns the test.
   getTestClass()   - 
Method in interface org.testng. ITestNGMethod 
  
   getTestClass()   - 
Method in interface org.testng. ITestResult 
  
   getTestClasses()   - 
Method in class org.testng. TestRunner 
  
   getTestContext()   - 
Method in interface org.testng. ISuiteResult 
  
   getTestContext()   - 
Method in interface org.testng. ITestResult 
  
   getTestContexts()   - 
Method in class org.testng. TestListenerAdapter 
  
   getTestListeners()   - 
Method in class org.testng. TestNG 
  
   getTestListeners()   - 
Method in class org.testng. TestRunner 
  
   getTestMethod()   - 
Method in interface org.testng. IInvokedMethod 
  
   getTestMethods()   - 
Method in interface org.testng. ITestClass 
 Returns all the applicable test methods.
   getTestMethods(Class, XmlTest)   - 
Method in interface org.testng. ITestMethodFinder 
  
   getTestName()   - 
Method in interface org.testng.annotations. ITestAnnotation 
  
   getTestName()   - 
Method in interface org.testng. IClass 
 If this class implements ITest, returns its test name, otherwise returns null.
   getTestName()   - 
Method in interface org.testng. ITest 
 The name of test instance(s).
   getTestName()   - 
Method in interface org.testng. ITestResult 
 If this result's related instance implements ITest, returns its test name, otherwise returns null.
   getTestParameters()   - 
Method in class org.testng.xml. XmlTest 
  
   getTestResult()   - 
Method in interface org.testng. IInvokedMethod 
  
   getTests()   - 
Method in class org.testng.xml. XmlSuite 
 Returns the tests.
   getThreadCount()   - 
Method in class org.testng.xml. XmlSuite 
 Returns the threadCount.
   getThreadCount()   - 
Method in class org.testng.xml. XmlTest 
  
   getThreadPoolSize()   - 
Method in interface org.testng.annotations. ITestAnnotation 
 The size of the thread pool for this method.
   getThreadPoolSize()   - 
Method in interface org.testng. ITestNGMethod 
  
   getThrowable()   - 
Method in interface org.testng. ITestResult 
  
   getTimeOut()   - 
Method in interface org.testng.annotations. ITestOrConfiguration 
 Returns the maximum number of milliseconds this test should take.
   getTimeOut()   - 
Method in interface org.testng. ITestNGMethod 
  
   getTimeOut()   - 
Method in class org.testng. SuiteRunnerWorker 
  
   getTimeOut()   - 
Method in class org.testng.xml. XmlSuite 
 Returns the timeout.
   getTimeOut(long)   - 
Method in class org.testng.xml. XmlSuite 
 Returns the timeout as a long value specifying the default value to be used if
 no timeout was specified.
   getTimeOut()   - 
Method in class org.testng.xml. XmlTest 
  
   getTimeOut(long)   - 
Method in class org.testng.xml. XmlTest 
  
   getUserData()   - 
Method in interface org.testng. IMethodSelectorContext 
  
   getValue()   - 
Method in interface org.testng.annotations. IExpectedExceptionsAnnotation 
 The list of exceptions expected to be thrown by this method.
   getValue()   - 
Method in interface org.testng.annotations. IParametersAnnotation 
 The list of variables used to fill the parameters of this method.
   getValue()   - 
Method in class org.testng. ReporterConfig.Property 
  
   getVerbose()   - 
Static method in class org.testng. TestRunner 
  
   getVerbose()   - 
Method in class org.testng.xml. XmlSuite 
 Returns the verbose.
   getVerbose()   - 
Method in class org.testng.xml. XmlTest 
  
   getXmlClass()   - 
Method in interface org.testng. IClass 
  
   getXmlClasses()   - 
Method in class org.testng.xml. XmlPackage 
  
   getXmlClasses()   - 
Method in class org.testng.xml. XmlTest 
  
   getXmlPackages()   - 
Method in class org.testng.xml. XmlSuite 
 Returns the XML packages.
   getXmlPackages()   - 
Method in class org.testng.xml. XmlTest 
  
   getXmlSuite()   - 
Method in interface org.testng. ISuite 
  
   getXmlSuite()   - 
Method in class org.testng. SuiteRunner 
  
   getXmlTest()   - 
Method in interface org.testng. IClass 
  
   getXmlTest()   - 
Method in interface org.testng. ITestNGMethod 
  
   GROUPS   - 
Static variable in class org.testng. CommandLineArgs 
  
   groups   - 
Variable in class org.testng. CommandLineArgs 
  
   Guice   - Annotation Type in  org.testng.annotations  This annotation specifies what Guice modules should be used to instantiate
 this test class. 
 
    
 H  
 
   handleOutput(String)   - 
Method in class org.testng. TestNGAntTask 
  
   HAS_FAILURE   - 
Static variable in class org.testng. TestNG 
  
   HAS_FSP   - 
Static variable in class org.testng. TestNG 
  
   HAS_NO_TEST   - 
Static variable in class org.testng. TestNG 
  
   HAS_SKIPPED   - 
Static variable in class org.testng. TestNG 
  
   hasFailure()   - 
Method in class org.testng. TestNG 
  
   hasFailureWithinSuccessPercentage()   - 
Method in class org.testng. TestNG 
  
   hashCode()   - 
Method in class org.testng.xml. XmlClass 
  
   hashCode()   - 
Method in class org.testng.xml. XmlInclude 
  
   hashCode()   - 
Method in class org.testng.xml. XmlMethodSelector 
  
   hashCode()   - 
Method in class org.testng.xml. XmlPackage 
  
   hashCode()   - 
Method in class org.testng.xml. XmlSuite 
  
   hashCode()   - 
Method in class org.testng.xml. XmlTest 
  
   hasSkip()   - 
Method in class org.testng. TestNG 
  
   HOST   - 
Static variable in class org.testng. CommandLineArgs 
  
   host   - 
Variable in class org.testng. CommandLineArgs 
  
 
 
    
 I  
 
   IAnnotation   - Interface in  org.testng.annotations  The parent interface for all the annotations.   IAnnotationTransformer   - Interface in  org.testng      IAnnotationTransformer2   - Interface in  org.testng  Use this interface instead of IAnnotationTransformer if you want to modify any TestNG
 annotation besides @Test.   IAttributes   - Interface in  org.testng  A trait that is used by all interfaces that lets the user add or remove their
 own attributes.   IClass   - Interface in  org.testng   IClass  represents a test class and a collection of its instances.   IConfigurable   - Interface in  org.testng  If a test class implements this interface, its run() method
 will be invoked instead of each configuration method found.   IConfigurationAnnotation   - Interface in  org.testng.annotations  Encapsulate the @Configuration / @testng.configuration annotation

 Created on Dec 20, 2005   IConfigurationListener   - Interface in  org.testng  Listener interface for events related to configuration methods.   IConfigurationListener2   - Interface in  org.testng      IConfigureCallBack   - Interface in  org.testng  A parameter of this type will be passed to the run() method of a IConfigurable.   IDataProviderAnnotation   - Interface in  org.testng.annotations  Encapsulate the @DataProvider / @testng.data-provider annotation

 Created on Dec 20, 2005   IExecutionListener   - Interface in  org.testng  A listener used to monitor when a TestNG run starts and ends.   IExpectedExceptionsAnnotation   - Interface in  org.testng.annotations  Encapsulate the @ExpectedExceptions / @testng.expected-exceptions annotation
 Created on Dec 20, 2005   IExtraOutput   - Interface in  org.testng  This class is used by Reporter to store the extra output to be later
 included in the HTML report:
 - User-generated report
 - Parameter info

 Created on Feb 16, 2006   IFactoryAnnotation   - Interface in  org.testng.annotations  Encapsulate the @Factory / @testng.factory annotation

 Created on Dec 20, 2005   ignoreMissingDependencies()   - 
Method in interface org.testng.annotations. ITestAnnotation 
  
   ignoreMissingDependencies()   - 
Method in interface org.testng. ITestNGMethod 
  
   IHookable   - Interface in  org.testng  If a test class implements this interface, its run() method
 will be invoked instead of each @Test method found.   IHookCallBack   - Interface in  org.testng  A parameter of this type will be passed to the run() method of a IHookable.   IInstanceInfo   - Interface in  org.testng  This class defines a pair of instance/class.   IInvokedMethod   - Interface in  org.testng  An interface representing a method that has been invoked by TestNG.   IInvokedMethodListener   - Interface in  org.testng  A listener that gets invoked before and after a method is invoked by TestNG.   IInvokedMethodListener2   - Interface in  org.testng  Implement this interface if you need a handle to   ITestContext  .   IMethodInstance   - Interface in  org.testng  This interface captures a test method along with all the instances it should
 be run on.   IMethodInterceptor   - Interface in  org.testng  This class is used to alter the list of test methods that TestNG is about to run.   IMethodSelector   - Interface in  org.testng  This interface is used to augment or replace TestNG's algorithm to
 decide whether a test method should be included in a test run.   IMethodSelectorContext   - Interface in  org.testng  An implementation of this interface is passed to all the Method Selectors
 when their includeMethod() is invoked.   IModuleFactory   - Interface in  org.testng  This interface is used by the moduleFactory attribute of the @Guice
 annotation.   includeMethod(IMethodSelectorContext, ITestNGMethod, boolean)   - 
Method in interface org.testng. IMethodSelector 
  
   incrementCurrentInvocationCount()   - 
Method in interface org.testng. ITestNGMethod 
  
   initializeSuitesAndJarFile()   - 
Method in class org.testng. TestNG 
  
   intercept(List<IMethodInstance>, ITestContext)   - 
Method in interface org.testng. IMethodInterceptor 
  
   invocationTimeOut()   - 
Method in interface org.testng.annotations. ITestAnnotation 
  
   IObjectFactory   - Interface in  org.testng  Factory used to create all test instances.   IObjectFactory2   - Interface in  org.testng  Factory used to create all test instances.   IObjectFactoryAnnotation   - Interface in  org.testng.annotations      IParameterizable   - Interface in  org.testng.annotations  Parent interface for annotations that can receive parameters.   IParametersAnnotation   - Interface in  org.testng.annotations  Encapsulate the @Parameters / @testng.parameters annotation

 Created on Dec 20, 2005   IReporter   - Interface in  org.testng  This interface can be implemented by clients to generate a report.   IResultMap   - Interface in  org.testng      IRetryAnalyzer   - Interface in  org.testng  Interface to implement to be able to have a chance to retry a failed test.   isAfterClassConfiguration()   - 
Method in interface org.testng. ITestNGMethod 
  
   isAfterGroupsConfiguration()   - 
Method in interface org.testng. ITestNGMethod 
  
   isAfterMethodConfiguration()   - 
Method in interface org.testng. ITestNGMethod 
  
   isAfterSuiteConfiguration()   - 
Method in interface org.testng. ITestNGMethod 
  
   isAfterTestConfiguration()   - 
Method in interface org.testng. ITestNGMethod 
  
   isAlwaysRun()   - 
Method in interface org.testng. ITestNGMethod 
  
   isBeforeClassConfiguration()   - 
Method in interface org.testng. ITestNGMethod 
  
   isBeforeGroupsConfiguration()   - 
Method in interface org.testng. ITestNGMethod 
  
   isBeforeMethodConfiguration()   - 
Method in interface org.testng. ITestNGMethod 
  
   isBeforeSuiteConfiguration()   - 
Method in interface org.testng. ITestNGMethod 
  
   isBeforeTestConfiguration()   - 
Method in interface org.testng. ITestNGMethod 
  
   isConfigurationMethod()   - 
Method in interface org.testng. IInvokedMethod 
  
   isFailed()   - 
Method in class org.testng. SuiteRunState 
  
   isFakeConfiguration()   - 
Method in interface org.testng.annotations. IConfigurationAnnotation 
 Internal use only.
   isJdk14()   - 
Static method in class org.testng. TestNG 
  Deprecated.  
   isJUnit()   - 
Method in class org.testng.xml. XmlSuite 
 Returns the JUnit compatibility flag.
   isJUnit()   - 
Method in class org.testng.xml. XmlTest 
  
   isParallel()   - 
Method in interface org.testng.annotations. IDataProviderAnnotation 
 Whether this data provider should be used in parallel.
   isSkip()   - 
Method in exception org.testng. SkipException 
 Flag if the current exception marks a skipped method ( true )
 or a failure ( false ).
   isSkip()   - 
Method in exception org.testng. TimeBombSkipException 
  
   isStopped()   - 
Method in interface org.testng. IMethodSelectorContext 
  
   isSuccess()   - 
Method in interface org.testng. ITestResult 
  
   isTest()   - 
Method in interface org.testng. ITestNGMethod 
  
   isTestMethod()   - 
Method in interface org.testng. IInvokedMethod 
  
   ISuite   - Interface in  org.testng  Interface defining a Test Suite.   ISuiteListener   - Interface in  org.testng  Listener for test suites.   ISuiteResult   - Interface in  org.testng  This class represents the result of a suite run.   ITest   - Interface in  org.testng  If a test class implements this interface, it will receive a
 special treatment, such as having the test name displayed
 in the HTML reports.   ITestAnnotation   - Interface in  org.testng.annotations  Encapsulate the @Test / @testng.test annotation.   ITestClass   - Interface in  org.testng  This class represents a test class:
 
  The test methods
 The configuration methods (test and method)
 The class file
 

 Note that the methods returned by instances of this class
 are expected to be correct at runtime.   ITestClassFinder   - Interface in  org.testng  This class is used by TestNG to locate the test classes.   ITestContext   - Interface in  org.testng  This class defines a test context which contains all the information
 for a given test run.   ITestListener   - Interface in  org.testng  A listener for test running.   ITestMethodFinder   - Interface in  org.testng  This interface allows to modify the strategy used by TestRunner
 to find its test methods.   ITestNGListener   - Interface in  org.testng  This is a marker interface for all objects that can be passed
 as a -listener argument.   ITestNGListenerFactory   - Interface in  org.testng  A factory used to create instances of ITestNGListener.   ITestNGMethod   - Interface in  org.testng  Describes a TestNG annotated method and the instance on which it will be invoked.   ITestObjectFactory   - Interface in  org.testng  Parent interface of all the object factories.   ITestOrConfiguration   - Interface in  org.testng.annotations  This interface captures methods common to @Test and @Configuration

 Created on Dec 20, 2005   ITestResult   - Interface in  org.testng  This class describes the result of a test.   ITestRunnerFactory   - Interface in  org.testng  A factory for TestRunners to be used by SuiteRunners. 
 
    
 J  
 
   JUNIT   - 
Static variable in class org.testng. CommandLineArgs 
  
   junit   - 
Variable in class org.testng. CommandLineArgs 
  
 
 
    
 L  
 
   LISTENER   - 
Static variable in class org.testng. CommandLineArgs 
  
   listener   - 
Variable in class org.testng. CommandLineArgs 
  
   Listeners   - Annotation Type in  org.testng.annotations  This annotation lets you define listeners directly on a test class
 instead of doing so in your testng.xml.   listToString(List<Integer>)   - 
Static method in class org.testng.xml. XmlClass 
  
   loadClasses()   - 
Method in class org.testng.xml. XmlClass 
  
   LOG   - 
Static variable in class org.testng. CommandLineArgs 
  
   log(String)   - 
Static method in class org.testng. Reporter 
 Log the passed string to the HTML reports
   log(String, int, boolean)   - 
Static method in class org.testng. Reporter 
 Log the passed string to the HTML reports if the current verbosity
 is equal or greater than the one passed in parameter.
   log(String, boolean)   - 
Static method in class org.testng. Reporter 
 Log the passed string to the HTML reports.
   log(String, int)   - 
Static method in class org.testng. Reporter 
 Log the passed string to the HTML reports if the current verbosity
 is equal or greater than the one passed in parameter
 
 
    
 M  
 
   m_assertEnabled   - 
Variable in class org.testng. TestNGAntTask 
  
   m_classFilesets   - 
Variable in class org.testng. TestNGAntTask 
  
   m_configFailurePolicy   - 
Variable in class org.testng. TestNGAntTask 
  
   m_dataproviderthreadCount   - 
Variable in class org.testng. TestNGAntTask 
  
   m_dump   - 
Variable in class org.testng. TestNGAntTask 
 True if the temporary file created by the Ant Task for command line parameters
 to TestNG should be preserved after execution.
   m_end   - 
Variable in class org.testng. TestNG 
  
   m_environment   - 
Variable in class org.testng. TestNGAntTask 
  
   m_excludedGroups   - 
Variable in class org.testng. TestNGAntTask 
  
   m_failurePropertyName   - 
Variable in class org.testng. TestNGAntTask 
  
   m_fspPropertyName   - 
Variable in class org.testng. TestNGAntTask 
  
   m_haltOnFailure   - 
Variable in class org.testng. TestNGAntTask 
  
   m_haltOnFSP   - 
Variable in class org.testng. TestNGAntTask 
  
   m_haltOnSkipped   - 
Variable in class org.testng. TestNGAntTask 
  
   m_includedGroups   - 
Variable in class org.testng. TestNGAntTask 
  
   m_javaCommand   - 
Variable in class org.testng. TestNGAntTask 
  
   m_mainClass   - 
Variable in class org.testng. TestNGAntTask 
 The suite runner name (defaults to TestNG.class.getName().
   m_onHaltTarget   - 
Variable in class org.testng. TestNGAntTask 
  
   m_outputDir   - 
Variable in class org.testng. TestNGAntTask 
  
   m_parallelMode   - 
Variable in class org.testng. TestNGAntTask 
  
   m_randomizeSuites   - 
Variable in class org.testng. TestNGAntTask 
  
   m_skippedPropertyName   - 
Variable in class org.testng. TestNGAntTask 
  
   m_start   - 
Variable in class org.testng. TestNG 
  
   m_suites   - 
Variable in class org.testng. TestNG 
  
   m_testjar   - 
Variable in class org.testng. TestNGAntTask 
  
   m_testRunnerFactory   - 
Variable in class org.testng. TestNGAntTask 
  
   m_threadCount   - 
Variable in class org.testng. TestNGAntTask 
  
   m_useDefaultListeners   - 
Variable in class org.testng. TestNG 
  
   m_useDefaultListeners   - 
Variable in class org.testng. TestNGAntTask 
  
   m_workingDir   - 
Variable in class org.testng. TestNGAntTask 
  
   m_xmlFilesets   - 
Variable in class org.testng. TestNGAntTask 
  
   main(String[])   - 
Static method in class org.testng. Converter 
  
   main(String[])   - 
Static method in class org.testng. TestNG 
 The TestNG entry point for command line execution.
   MASTER   - 
Static variable in class org.testng. CommandLineArgs 
  
   master   - 
Variable in class org.testng. CommandLineArgs 
  
   METHOD_SELECTORS   - 
Static variable in class org.testng. CommandLineArgs 
  
   METHODS   - 
Static variable in class org.testng. CommandLineArgs 
  
   methodSelectors   - 
Variable in class org.testng. CommandLineArgs 
  
   MIXED   - 
Static variable in class org.testng. CommandLineArgs 
  
   mixed   - 
Variable in class org.testng. CommandLineArgs 
  
 
 
    
 N  
 
   newInstance(Constructor, Object...)   - 
Method in interface org.testng. IObjectFactory 
  
   newInstance(Class<?>)   - 
Method in interface org.testng. IObjectFactory2 
  
   newReporterInstance()   - 
Method in class org.testng. ReporterConfig 
 Creates a reporter based on the current configuration
   newTestRunner(ISuite, XmlTest, List<IInvokedMethodListener>)   - 
Method in interface org.testng. ITestRunnerFactory 
  
   NoInjection   - Annotation Type in  org.testng.annotations  Turn off TestNG injection for a parameter. 
 
    
 O  
 
   OBJECT_FACTORY   - 
Static variable in class org.testng. CommandLineArgs 
  
   ObjectFactory   - Annotation Type in  org.testng.annotations  Marks a method as the object factory to use for creating all test instances.   objectFactory   - 
Variable in class org.testng. CommandLineArgs 
  
   onConfigurationFailure(ITestResult)   - 
Method in interface org.testng. IConfigurationListener 
 Invoked whenever a configuration method failed.
   onConfigurationFailure(ITestResult)   - 
Method in class org.testng. TestListenerAdapter 
  
   onConfigurationFailure(ITestResult)   - 
Method in class org.testng. TestNG.ExitCodeListener 
  
   onConfigurationSkip(ITestResult)   - 
Method in interface org.testng. IConfigurationListener 
 Invoked whenever a configuration method was skipped.
   onConfigurationSkip(ITestResult)   - 
Method in class org.testng. TestListenerAdapter 
  
   onConfigurationSkip(ITestResult)   - 
Method in class org.testng. TestNG.ExitCodeListener 
  
   onConfigurationSuccess(ITestResult)   - 
Method in interface org.testng. IConfigurationListener 
 Invoked whenever a configuration method succeeded.
   onConfigurationSuccess(ITestResult)   - 
Method in class org.testng. TestListenerAdapter 
  
   onConfigurationSuccess(ITestResult)   - 
Method in class org.testng. TestNG.ExitCodeListener 
  
   onExecutionFinish()   - 
Method in interface org.testng. IExecutionListener 
 Invoked once all the suites have been run.
   onExecutionStart()   - 
Method in interface org.testng. IExecutionListener 
 Invoked before the TestNG run starts.
   onFinish(ISuite)   - 
Method in interface org.testng. ISuiteListener 
 This method is invoked after the SuiteRunner has run all
 the test suites.
   onFinish(ITestContext)   - 
Method in interface org.testng. ITestListener 
 Invoked after all the tests have run and all their
 Configuration methods have been called.
   onFinish(ITestContext)   - 
Method in class org.testng. TestListenerAdapter 
  
   onFinish(ITestContext)   - 
Method in class org.testng. TestNG.ExitCodeListener 
  
   onStart(ISuite)   - 
Method in interface org.testng. ISuiteListener 
 This method is invoked before the SuiteRunner starts.
   onStart(ITestContext)   - 
Method in interface org.testng. ITestListener 
 Invoked after the test class is instantiated and before
 any configuration method is called.
   onStart(ITestContext)   - 
Method in class org.testng. TestListenerAdapter 
  
   onStart(ITestContext)   - 
Method in class org.testng. TestNG.ExitCodeListener 
  
   onTestFailedButWithinSuccessPercentage(ITestResult)   - 
Method in interface org.testng. ITestListener 
 Invoked each time a method fails but has been annotated with
 successPercentage and this failure still keeps it within the
 success percentage requested.
   onTestFailedButWithinSuccessPercentage(ITestResult)   - 
Method in class org.testng. TestListenerAdapter 
  
   onTestFailedButWithinSuccessPercentage(ITestResult)   - 
Method in class org.testng. TestNG.ExitCodeListener 
  
   onTestFailure(ITestResult)   - 
Method in interface org.testng. ITestListener 
 Invoked each time a test fails.
   onTestFailure(ITestResult)   - 
Method in class org.testng. TestListenerAdapter 
  
   onTestFailure(ITestResult)   - 
Method in class org.testng. TestNG.ExitCodeListener 
  
   onTestSkipped(ITestResult)   - 
Method in interface org.testng. ITestListener 
 Invoked each time a test is skipped.
   onTestSkipped(ITestResult)   - 
Method in class org.testng. TestListenerAdapter 
  
   onTestSkipped(ITestResult)   - 
Method in class org.testng. TestNG.ExitCodeListener 
  
   onTestStart(ITestResult)   - 
Method in interface org.testng. ITestListener 
 Invoked each time before a test will be invoked.
   onTestStart(ITestResult)   - 
Method in class org.testng. TestListenerAdapter 
  
   onTestStart(ITestResult)   - 
Method in class org.testng. TestNG.ExitCodeListener 
  
   onTestSuccess(ITestResult)   - 
Method in interface org.testng. ITestListener 
 Invoked each time a test succeeds.
   onTestSuccess(ITestResult)   - 
Method in class org.testng. TestListenerAdapter 
  
   onTestSuccess(ITestResult)   - 
Method in class org.testng. TestNG.ExitCodeListener 
  
   Optional   - Annotation Type in  org.testng.annotations  Specifies that the current parameter is optional.   org.testng   - package org.testng     org.testng.annotations   - package org.testng.annotations     org.testng.xml   - package org.testng.xml     OUTPUT_DIRECTORY   - 
Static variable in class org.testng. CommandLineArgs 
  
   outputDirectory   - 
Variable in class org.testng. CommandLineArgs 
  
 
 
    
 P  
 
   PARALLEL   - 
Static variable in class org.testng. CommandLineArgs 
  
   PARALLEL_CLASSES   - 
Static variable in class org.testng.xml. XmlSuite 
  
   PARALLEL_INSTANCES   - 
Static variable in class org.testng.xml. XmlSuite 
  
   PARALLEL_METHODS   - 
Static variable in class org.testng.xml. XmlSuite 
  
   PARALLEL_MODES   - 
Static variable in class org.testng.xml. XmlSuite 
  
   PARALLEL_NONE   - 
Static variable in class org.testng.xml. XmlSuite 
  
   PARALLEL_TESTS   - 
Static variable in class org.testng.xml. XmlSuite 
 Parallel modes
   parallelMode   - 
Variable in class org.testng. CommandLineArgs 
  
   Parameters   - Annotation Type in  org.testng.annotations  Describes how to pass parameters to a @Test method.   PORT   - 
Static variable in class org.testng. CommandLineArgs 
  
   port   - 
Variable in class org.testng. CommandLineArgs 
  
   ppp(String)   - 
Static method in class org.testng. SuiteRunner 
  
   printStackTrace(PrintStream)   - 
Method in exception org.testng. TimeBombSkipException 
  
   printStackTrace(PrintWriter)   - 
Method in exception org.testng. TimeBombSkipException 
  
   privateMain(String[], ITestListener)   - 
Static method in class org.testng. TestNG 
  Note : this method is not part of the public API and is meant for internal usage only.
 
 
    
 R  
 
   RANDOMIZE_SUITES   - 
Static variable in class org.testng. CommandLineArgs 
  
   randomizeSuites   - 
Variable in class org.testng. CommandLineArgs 
  
   reduceStackTrace()   - 
Method in exception org.testng. SkipException 
 Subclasses may use this method to reduce the printed stack trace.
   removeAndCheckIfLast(ITestNGMethod, Object)   - 
Method in class org.testng. ClassMethodMap 
 Remove the method from this map and returns true if it is the last
 of its class.
   removeAttribute(String)   - 
Method in interface org.testng. IAttributes 
 Remove the attribute
   removeAttribute(String)   - 
Method in class org.testng. SuiteRunner 
  
   removeAttribute(String)   - 
Method in class org.testng. TestRunner 
  
   removeResult(ITestNGMethod)   - 
Method in interface org.testng. IResultMap 
  
   REPORTER   - 
Static variable in class org.testng. CommandLineArgs 
  
   reporter   - 
Variable in class org.testng. CommandLineArgs 
  
   Reporter   - Class in  org.testng  This class is used for test methods to log messages that will be
 included in the HTML reports generated by TestNG.   Reporter()   - 
Constructor for class org.testng. Reporter 
  
   ReporterConfig   - Class in  org.testng  Stores the information regarding the configuration of a pluggable report listener.   ReporterConfig()   - 
Constructor for class org.testng. ReporterConfig 
  
   ReporterConfig.Property   - Class in  org.testng      ReporterConfig.Property()   - 
Constructor for class org.testng. ReporterConfig.Property 
  
   restoreStackTrace()   - 
Method in exception org.testng. SkipException 
 Restores the original exception stack trace after a
 previous call to   SkipException.reduceStackTrace()  .
   retry(ITestResult)   - 
Method in interface org.testng. IRetryAnalyzer 
 Returns true if the test method has to be retried, false otherwise.
   run(IConfigureCallBack, ITestResult)   - 
Method in interface org.testng. IConfigurable 
  
   run(IHookCallBack, ITestResult)   - 
Method in interface org.testng. IHookable 
  
   run()   - 
Method in interface org.testng. ISuite 
 Triggers the start of running tests included in the suite.
   run()   - 
Method in class org.testng. SuiteRunner 
  
   run()   - 
Method in class org.testng. SuiteRunnerWorker 
  
   run()   - 
Method in class org.testng. TestNG 
 Run TestNG.
   run()   - 
Method in class org.testng. TestRunner 
 The main entry method for TestRunner.
   runConfigurationMethod(ITestResult)   - 
Method in interface org.testng. IConfigureCallBack 
 Invoke the test method currently being hijacked.
   runSuitesLocally()   - 
Method in class org.testng. TestNG 
 This needs to be public for maven2, for now..At least
 until an alternative mechanism is found.
   runTestMethod(ITestResult)   - 
Method in interface org.testng. IHookCallBack 
 Invoke the test method currently being hijacked.
 
 
    
 S  
 
   serialize()   - 
Method in class org.testng. ReporterConfig 
  
   setAllowReturnValues(Boolean)   - 
Method in class org.testng.xml. XmlSuite 
  
   setAllowReturnValues(Boolean)   - 
Method in class org.testng.xml. XmlTest 
  
   setAllTestMethods(List<ITestNGMethod>)   - 
Method in class org.testng. TestListenerAdapter 
  
   setAlwaysRun(boolean)   - 
Method in interface org.testng.annotations. ITestAnnotation 
  
   setAnnotationTransformer(IAnnotationTransformer)   - 
Method in class org.testng. TestNG 
  
   setAttribute(String, Object)   - 
Method in interface org.testng. IAttributes 
 Set a custom attribute.
   setAttribute(String, Object)   - 
Method in class org.testng. SuiteRunner 
  
   setAttribute(String, Object)   - 
Method in class org.testng. TestRunner 
  
   setBeanShellExpression(String)   - 
Method in class org.testng.xml. XmlTest 
  
   setClass(Class)   - 
Method in class org.testng.xml. XmlClass 
  
   setClasses(List<XmlClass>)   - 
Method in class org.testng.xml. XmlTest 
  
   setClassfilesetRef(Reference)   - 
Method in class org.testng. TestNGAntTask 
  
   setClassName(String)   - 
Method in class org.testng. ReporterConfig 
  
   setClassName(String)   - 
Method in class org.testng.xml. XmlMethodSelector 
  
   setClassNames(List<XmlClass>)   - 
Method in class org.testng.xml. XmlTest 
  Deprecated.   use setXmlClasses 
   setClasspath(Path)   - 
Method in class org.testng. TestNGAntTask 
 Set the classpath to be used when running the Java class
   setClasspathRef(Reference)   - 
Method in class org.testng. TestNGAntTask 
 Classpath to use, by reference.
   setCommandLineSuite(XmlSuite)   - 
Method in class org.testng. TestNG 
  
   setConfigFailurePolicy(String)   - 
Method in class org.testng. TestNG 
 Sets the policy for whether or not to ever invoke a configuration method again after
 it has failed once.
   setConfigFailurePolicy(String)   - 
Method in class org.testng. TestNGAntTask 
  
   setConfigFailurePolicy(String)   - 
Method in class org.testng.xml. XmlSuite 
 Sets the configuration failure policy.
   setCurrentTestResult(ITestResult)   - 
Static method in class org.testng. Reporter 
  
   setDataProvider(String)   - 
Method in interface org.testng.annotations. ITestAnnotation 
  
   setDataProviderClass(Class<?>)   - 
Method in interface org.testng.annotations. ITestAnnotation 
  
   setDataProviderThreadCount(int)   - 
Method in class org.testng. TestNG 
  
   setDataProviderThreadCount(String)   - 
Method in class org.testng. TestNGAntTask 
  
   setDataProviderThreadCount(int)   - 
Method in class org.testng.xml. XmlSuite 
  
   setDate(long)   - 
Method in interface org.testng. ITestNGMethod 
  
   setDefaultSuiteName(String)   - 
Method in class org.testng. TestNG 
  
   setDefaultTestName(String)   - 
Method in class org.testng. TestNG 
  
   setDelegateCommandSystemProperties(boolean)   - 
Method in class org.testng. TestNGAntTask 
  
   setDependsOnGroups(String[])   - 
Method in interface org.testng.annotations. ITestOrConfiguration 
  
   setDependsOnMethods(String[])   - 
Method in interface org.testng.annotations. ITestOrConfiguration 
  
   setDescription(String)   - 
Method in interface org.testng.annotations. ITestOrConfiguration 
  
   setDescription(String)   - 
Method in class org.testng.xml. XmlInclude 
  
   setDumpCommand(boolean)   - 
Method in class org.testng. TestNGAntTask 
 Sets the flag to log the command line.
   setDumpEnv(boolean)   - 
Method in class org.testng. TestNGAntTask 
 Sets the flag to write on  System.out  the Ant
 Environment properties.
   setDumpSys(boolean)   - 
Method in class org.testng. TestNGAntTask 
 Sets te flag to write on  System.out  the system properties.
   setEnableAssert(boolean)   - 
Method in class org.testng. TestNGAntTask 
  
   setEnabled(boolean)   - 
Method in interface org.testng.annotations. IParameterizable 
  
   setEndMillis(long)   - 
Method in interface org.testng. ITestResult 
  
   setExclude(List<String>)   - 
Method in class org.testng.xml. XmlPackage 
  
   setExcludedGroups(String)   - 
Method in class org.testng. TestNG 
 Define which groups will be excluded from this run.
   setExcludedGroups(String)   - 
Method in class org.testng. TestNGAntTask 
  
   setExcludedGroups(List<String>)   - 
Method in class org.testng.xml. XmlSuite 
  
   setExcludedGroups(List<String>)   - 
Method in class org.testng.xml. XmlTest 
  
   setExcludedMethods(List<String>)   - 
Method in class org.testng.xml. XmlClass 
  
   setExpectedExceptions(Class<?>[])   - 
Method in interface org.testng.annotations. ITestAnnotation 
  
   setExpectedExceptionsMessageRegExp(String)   - 
Method in interface org.testng.annotations. ITestAnnotation 
  
   setExpression(String)   - 
Method in class org.testng.xml. XmlMethodSelector 
  
   setExpression(String)   - 
Method in class org.testng.xml. XmlTest 
  
   setFailedButWithinSuccessPercentageTests(List<ITestResult>)   - 
Method in class org.testng. TestListenerAdapter 
  
   setFailedTests(List<ITestResult>)   - 
Method in class org.testng. TestListenerAdapter 
  
   setFailureProperty(String)   - 
Method in class org.testng. TestNGAntTask 
  
   setFileName(String)   - 
Method in class org.testng.xml. XmlSuite 
  
   setFSPProperty(String)   - 
Method in class org.testng. TestNGAntTask 
  
   setGroupByInstances(boolean)   - 
Method in class org.testng. TestNG 
  
   setGroupByInstances(boolean)   - 
Method in class org.testng.xml. XmlSuite 
  
   setGroupByInstances(boolean)   - 
Method in class org.testng.xml. XmlTest 
  
   setGroups(String[])   - 
Method in interface org.testng.annotations. ITestOrConfiguration 
  
   setGroups(String)   - 
Method in class org.testng. TestNG 
 Define which groups will be included from this run.
   setGroups(String)   - 
Method in class org.testng. TestNGAntTask 
  
   setHaltonfailure(boolean)   - 
Method in class org.testng. TestNGAntTask 
  
   setHaltonFSP(boolean)   - 
Method in class org.testng. TestNGAntTask 
  
   setHaltonskipped(boolean)   - 
Method in class org.testng. TestNGAntTask 
  
   setHasFailure(boolean)   - 
Method in class org.testng. TestNG 
  Deprecated.   since 5.1 
   setHasFailureWithinSuccessPercentage(boolean)   - 
Method in class org.testng. TestNG 
  Deprecated.   since 5.1 
   setHasSkip(boolean)   - 
Method in class org.testng. TestNG 
  Deprecated.   since 5.1 
   setHost(String)   - 
Method in class org.testng. SuiteRunner 
  
   setId(String)   - 
Method in interface org.testng. ITestNGMethod 
  
   setIgnoreMissingDependencies(boolean)   - 
Method in interface org.testng.annotations. ITestAnnotation 
  
   setIgnoreMissingDependencies(boolean)   - 
Method in interface org.testng. ITestNGMethod 
  
   setInclude(List<String>)   - 
Method in class org.testng.xml. XmlPackage 
  
   setIncludedGroups(List<String>)   - 
Method in class org.testng.xml. XmlSuite 
  
   setIncludedGroups(List<String>)   - 
Method in class org.testng.xml. XmlTest 
  
   setIncludedMethods(List<XmlInclude>)   - 
Method in class org.testng.xml. XmlClass 
  
   setIndex(int)   - 
Method in class org.testng.xml. XmlClass 
  
   setInvocationCount(int)   - 
Method in interface org.testng.annotations. ITestAnnotation 
  
   setInvocationCount(int)   - 
Method in interface org.testng. ITestNGMethod 
  
   setInvocationNumbers(List<Integer>)   - 
Method in interface org.testng. ITestNGMethod 
  
   setInvocationTimeOut(long)   - 
Method in interface org.testng.annotations. ITestAnnotation 
  
   setJUnit(Boolean)   - 
Method in class org.testng. TestNG 
 Specify if this run should be made in JUnit mode
   setJUnit(boolean)   - 
Method in class org.testng. TestNGAntTask 
  
   setJUnit(Boolean)   - 
Method in class org.testng.xml. XmlSuite 
 Sets the JUnit compatibility flag.
   setJunit(Boolean)   - 
Method in class org.testng.xml. XmlSuite 
  
   setJUnit(boolean)   - 
Method in class org.testng.xml. XmlTest 
  
   setJunit(boolean)   - 
Method in class org.testng.xml. XmlTest 
  
   setJvm(String)   - 
Method in class org.testng. TestNGAntTask 
 Sets a particular JVM to be used.
   setLanguage(String)   - 
Method in class org.testng.xml. XmlMethodSelector 
  
   setListener(String)   - 
Method in class org.testng. TestNGAntTask 
  Deprecated.   Use "listeners" 
   setListenerClasses(List<Class>)   - 
Method in class org.testng. TestNG 
 Define which listeners to user for this run.
   setListeners(String)   - 
Method in class org.testng. TestNGAntTask 
  
   setListeners(List<String>)   - 
Method in class org.testng.xml. XmlSuite 
  
   setMaster(String)   - 
Method in class org.testng. TestNG 
 Specify if this run should be in Master-Slave mode as Master
   setMetaGroups(Map<String, List<String>>)   - 
Method in class org.testng.xml. XmlTest 
  
   setMethodInterceptor(IMethodInterceptor)   - 
Method in class org.testng. TestNG 
  
   setMethodInterceptor(IMethodInterceptor)   - 
Method in class org.testng. TestRunner 
  
   setMethods(String)   - 
Method in class org.testng. TestNGAntTask 
  
   setMethodSelectors(String)   - 
Method in class org.testng. TestNGAntTask 
  
   setMethodSelectors(List<XmlMethodSelector>)   - 
Method in class org.testng.xml. XmlSuite 
 Sets the method selectors.
   setMethodSelectors(List<XmlMethodSelector>)   - 
Method in class org.testng.xml. XmlTest 
  
   setMissingGroup(String)   - 
Method in interface org.testng. ITestNGMethod 
  
   setMixed(Boolean)   - 
Method in class org.testng. TestNG 
 Specify if this run should be made in mixed mode
   setMode(TestNGAntTask.Mode)   - 
Method in class org.testng. TestNGAntTask 
  
   setName(String)   - 
Method in interface org.testng.annotations. IDataProviderAnnotation 
  
   setName(String)   - 
Method in class org.testng. ReporterConfig.Property 
  
   setName(String)   - 
Method in class org.testng.xml. XmlClass 
  
   setName(String)   - 
Method in class org.testng.xml. XmlMethodSelector 
  
   setName(String)   - 
Method in class org.testng.xml. XmlPackage 
  
   setName(String)   - 
Method in class org.testng.xml. XmlSuite 
 Sets the name.
   setName(String)   - 
Method in class org.testng.xml. XmlTest 
  
   setObjectFactory(ITestObjectFactory)   - 
Method in class org.testng. SuiteRunner 
  
   setObjectFactory(Class)   - 
Method in class org.testng. TestNG 
  
   setObjectFactory(ITestObjectFactory)   - 
Method in class org.testng. TestNG 
  
   setObjectFactory(String)   - 
Method in class org.testng. TestNGAntTask 
  
   setObjectFactory(ITestObjectFactory)   - 
Method in class org.testng.xml. XmlSuite 
  
   setOnHaltTarget(String)   - 
Method in class org.testng. TestNGAntTask 
  
   setOutputDir(File)   - 
Method in class org.testng. TestNGAntTask 
 Sets the test output directory
   setOutputDirectory(String)   - 
Method in class org.testng. TestNG 
 Sets the output directory where the reports will be created.
   setOutputDirectory(String)   - 
Method in class org.testng. TestRunner 
  
   setPackages(List<XmlPackage>)   - 
Method in class org.testng.xml. XmlSuite 
  
   setPackages(List<XmlPackage>)   - 
Method in class org.testng.xml. XmlTest 
  
   setParallel(boolean)   - 
Method in interface org.testng.annotations. IDataProviderAnnotation 
  
   setParallel(String)   - 
Method in class org.testng. TestNG 
 Define whether this run will be run in parallel mode.
   setParallel(String)   - 
Method in class org.testng. TestNGAntTask 
  
   setParallel(String)   - 
Method in class org.testng.xml. XmlSuite 
 Sets the parallel mode
   setParallel(String)   - 
Method in class org.testng.xml. XmlTest 
  
   setParameterInvocationCount(int)   - 
Method in interface org.testng. ITestNGMethod 
  
   setParameters(Object[])   - 
Method in interface org.testng. ITestResult 
  
   setParameters(Map<String, String>)   - 
Method in class org.testng.xml. XmlInclude 
  
   setParameters(Map<String, String>)   - 
Method in class org.testng.xml. XmlSuite 
 Sets parameters.
   setParameters(Map<String, String>)   - 
Method in class org.testng.xml. XmlTest 
  
   setParentSuite(XmlSuite)   - 
Method in class org.testng.xml. XmlSuite 
  
   setPassedTests(List<ITestResult>)   - 
Method in class org.testng. TestListenerAdapter 
  
   setPreserveOrder(boolean)   - 
Method in class org.testng. TestNG 
  
   setPreserveOrder(String)   - 
Method in class org.testng.xml. XmlSuite 
  
   setPreserveOrder(String)   - 
Method in class org.testng.xml. XmlTest 
  
   setPriority(int)   - 
Method in interface org.testng.annotations. ITestAnnotation 
  
   setPriority(int)   - 
Method in interface org.testng. ITestNGMethod 
  
   setPriority(int)   - 
Method in class org.testng.xml. XmlMethodSelector 
  
   setProperty(Properties, String, String, String)   - 
Static method in class org.testng.xml. XmlUtils 
 Don't add this property if it's equal to its default value.
   setRandomizeSuites(boolean)   - 
Method in class org.testng. TestNG 
  
   setRandomizeSuites(Boolean)   - 
Method in class org.testng. TestNGAntTask 
  
   setReporter(String)   - 
Method in class org.testng. TestNGAntTask 
  
   setReportResults(boolean)   - 
Method in class org.testng. SuiteRunner 
  
   setRetryAnalyzer(Class<?>)   - 
Method in interface org.testng.annotations. ITestAnnotation 
  
   setRetryAnalyzer(IRetryAnalyzer)   - 
Method in interface org.testng. ITestNGMethod 
  
   setSequential(boolean)   - 
Method in interface org.testng.annotations. ITestAnnotation 
  
   setServiceLoaderClassLoader(URLClassLoader)   - 
Method in class org.testng. TestNG 
  
   setSingleThreaded(boolean)   - 
Method in interface org.testng.annotations. ITestAnnotation 
  
   setSkipFailedInvocationCounts(Boolean)   - 
Method in class org.testng. SuiteRunner 
  
   setSkipFailedInvocationCounts(Boolean)   - 
Method in class org.testng. TestNG 
  
   setSkipFailedInvocationCounts(boolean)   - 
Method in class org.testng. TestNGAntTask 
  
   setSkipFailedInvocationCounts(boolean)   - 
Method in class org.testng.xml. XmlSuite 
  
   setSkipFailedInvocationCounts(boolean)   - 
Method in class org.testng.xml. XmlTest 
  
   setSkipFailedInvocations(boolean)   - 
Method in interface org.testng.annotations. ITestAnnotation 
  
   setSkipFailedInvocations(boolean)   - 
Method in interface org.testng. ITestNGMethod 
  
   setSkippedProperty(String)   - 
Method in class org.testng. TestNGAntTask 
  
   setSkippedTests(List<ITestResult>)   - 
Method in class org.testng. TestListenerAdapter 
  
   setSlave(String)   - 
Method in class org.testng. TestNG 
 Specify if this run should be in Master-Slave mode as slave
   setSourcePath(String)   - 
Method in class org.testng. TestNG 
 This method is invoked by Maven's Surefire, only remove it once
 Surefire has been modified to no longer call it.
   setStatus(int)   - 
Method in interface org.testng. ITestResult 
  
   setStopped(boolean)   - 
Method in interface org.testng. IMethodSelectorContext 
 Indicate that no other Method Selectors should be invoked after the
 current one if stopped is false.
   setSuccessPercentage(int)   - 
Method in interface org.testng.annotations. ITestAnnotation 
  
   setSuite(XmlSuite)   - 
Method in class org.testng.xml. XmlTest 
  
   setSuiteFiles(List<String>)   - 
Method in class org.testng.xml. XmlSuite 
 Sets the suite files.
   setSuiteName(String)   - 
Method in interface org.testng.annotations. ITestAnnotation 
  
   setSuiteName(String)   - 
Method in class org.testng. TestNGAntTask 
 Sets the suite name
   setSuiteRunnerClass(String)   - 
Method in class org.testng. TestNGAntTask 
 Sets the suite runner class to invoke
   setSuiteThreadPoolSize(Integer)   - 
Method in class org.testng. TestNG 
  
   setSuiteThreadPoolSize(Integer)   - 
Method in class org.testng. TestNGAntTask 
  
   setTestClass(ITestClass)   - 
Method in interface org.testng. ITestNGMethod 
 Sets the test class having this method.
   setTestClasses(Class[])   - 
Method in class org.testng. TestNG 
 Set the test classes to be run by this TestNG object.
   setTestJar(String)   - 
Method in class org.testng. TestNG 
 Sets a jar containing a testng.xml file.
   setTestJar(File)   - 
Method in class org.testng. TestNGAntTask 
 Sets the test jar
   setTestMethods(List<ITestNGMethod>)   - 
Method in interface org.testng. IMethodSelector 
 Invoked when all the test methods are known so that the method selector
 can perform additional work, such as adding the transitive closure of
 all the groups being included and depended upon.
   setTestName(String)   - 
Method in interface org.testng.annotations. ITestAnnotation 
  
   setTestName(String)   - 
Method in class org.testng. TestNGAntTask 
 Sets the test name
   setTestName(String)   - 
Method in class org.testng. TestRunner 
  
   setTestNames(List<String>)   - 
Method in class org.testng. TestNG 
 Only run the specified tests from the suite.
   setTestNames(String)   - 
Method in class org.testng. TestNGAntTask 
  
   setTestNGVersion()   - 
Static method in class org.testng. TestNG 
  Deprecated.   The TestNG version is now established at load time. This
 method is not required anymore and is now a no-op. 
   setTestRunnerFactory(ITestRunnerFactory)   - 
Method in class org.testng. TestNG 
  
   setTestRunnerFactory(String)   - 
Method in class org.testng. TestNGAntTask 
  
   setTests(List<XmlTest>)   - 
Method in class org.testng.xml. XmlSuite 
  
   setTestSuites(List<String>)   - 
Method in class org.testng. TestNG 
 Set the suites file names to be run by this TestNG object.
   setThreadCount(int)   - 
Method in class org.testng. TestNG 
 Define the number of threads in the thread pool.
   setThreadCount(String)   - 
Method in class org.testng. TestNGAntTask 
  
   setThreadCount(int)   - 
Method in class org.testng.xml. XmlSuite 
 Set the thread count.
   setThreadCount(int)   - 
Method in class org.testng.xml. XmlTest 
  
   setThreadPoolSize(int)   - 
Method in interface org.testng.annotations. ITestAnnotation 
  
   setThreadPoolSize(int)   - 
Method in interface org.testng. ITestNGMethod 
  
   setThrowable(Throwable)   - 
Method in interface org.testng. ITestResult 
  
   setTimeOut(long)   - 
Method in interface org.testng.annotations. ITestOrConfiguration 
  
   setTimeOut(long)   - 
Method in interface org.testng. ITestNGMethod 
  
   setTimeout(Integer)   - 
Method in class org.testng. TestNGAntTask 
 Set the timeout value (in milliseconds).
   setTimeOut(String)   - 
Method in class org.testng.xml. XmlSuite 
 Sets the timeout.
   setTimeOut(long)   - 
Method in class org.testng.xml. XmlTest 
  
   setUseDefaultListeners(boolean)   - 
Method in class org.testng. TestNG 
 If this method is passed true before run(), the default listeners
 will not be used.
   setUseDefaultListeners(String)   - 
Method in class org.testng. TestNGAntTask 
  
   setValue(String)   - 
Method in class org.testng. ReporterConfig.Property 
  
   setVerbose(int)   - 
Method in class org.testng. TestNG 
 Sets the level of verbosity.
   setVerbose(Integer)   - 
Method in class org.testng. TestNGAntTask 
  
   setVerbose(int)   - 
Method in class org.testng. TestRunner 
  
   setVerbose(Integer)   - 
Method in class org.testng.xml. XmlSuite 
 Set the verbose.
   setVerbose(int)   - 
Method in class org.testng.xml. XmlTest 
  
   setWorkingDir(File)   - 
Method in class org.testng. TestNGAntTask 
 The directory to invoke the VM in.
   setXmlClasses(List<XmlClass>)   - 
Method in class org.testng.xml. XmlTest 
 Sets the XML Classes.
   setXmlfilesetRef(Reference)   - 
Method in class org.testng. TestNGAntTask 
  
   setXmlPackages(List<XmlPackage>)   - 
Method in class org.testng.xml. XmlSuite 
 Sets the XML packages.
   setXmlPackages(List<XmlPackage>)   - 
Method in class org.testng.xml. XmlTest 
  
   setXmlPathInJar(String)   - 
Method in class org.testng. TestNG 
 Sets the path to the XML file in the test jar file.
   setXmlPathInJar(String)   - 
Method in class org.testng. TestNGAntTask 
  
   setXmlSuites(List<XmlSuite>)   - 
Method in class org.testng. TestNG 
 Specifies the XmlSuite objects to run.
   SHOW_TESTNG_STACK_FRAMES   - 
Static variable in class org.testng. TestNG 
 System properties
   size()   - 
Method in interface org.testng. IResultMap 
  
   SKIP   - 
Static variable in interface org.testng. ITestResult 
  
   SKIP   - 
Static variable in class org.testng.xml. XmlSuite 
 Configuration failure policy options
   SKIP_FAILED_INVOCATION_COUNTS   - 
Static variable in class org.testng. CommandLineArgs 
  
   SkipException   - Exception in  org.testng  The root exception for special skip handling.   SkipException(String)   - 
Constructor for exception org.testng. SkipException 
  
   SkipException(String, Throwable)   - 
Constructor for exception org.testng. SkipException 
  
   skipFailedInvocationCounts   - 
Variable in class org.testng. CommandLineArgs 
  
   skipFailedInvocationCounts()   - 
Method in class org.testng.xml. XmlSuite 
  
   skipFailedInvocationCounts()   - 
Method in class org.testng.xml. XmlTest 
  
   skipFailedInvocations()   - 
Method in interface org.testng.annotations. ITestAnnotation 
  
   skipFailedInvocations()   - 
Method in interface org.testng. ITestNGMethod 
  
   SLAVE   - 
Static variable in class org.testng. CommandLineArgs 
  
   slave   - 
Variable in class org.testng. CommandLineArgs 
  
   STARTED   - 
Static variable in interface org.testng. ITestResult 
  
   SUCCESS   - 
Static variable in interface org.testng. ITestResult 
  
   SUCCESS_PERCENTAGE_FAILURE   - 
Static variable in interface org.testng. ITestResult 
  
   SUITE_NAME   - 
Static variable in class org.testng. CommandLineArgs 
  
   SUITE_THREAD_POOL_SIZE   - 
Static variable in class org.testng. CommandLineArgs 
  
   SUITE_THREAD_POOL_SIZE_DEFAULT   - 
Static variable in class org.testng. CommandLineArgs 
  
   suiteFiles   - 
Variable in class org.testng. CommandLineArgs 
  
   suiteName   - 
Variable in class org.testng. CommandLineArgs 
  
   SuiteRunner   - Class in  org.testng   SuiteRunner  is responsible for running all the tests included in one
 suite.   SuiteRunner(IConfiguration, XmlSuite, String)   - 
Constructor for class org.testng. SuiteRunner 
  
   SuiteRunner(IConfiguration, XmlSuite, String, ITestRunnerFactory)   - 
Constructor for class org.testng. SuiteRunner 
  
   SuiteRunner(IConfiguration, XmlSuite, String, ITestRunnerFactory, boolean)   - 
Constructor for class org.testng. SuiteRunner 
  
   SuiteRunner(IConfiguration, XmlSuite, String, ITestRunnerFactory, boolean, IMethodInterceptor, List<IInvokedMethodListener>, List<ITestListener>)   - 
Constructor for class org.testng. SuiteRunner 
  
   SuiteRunnerWorker   - Class in  org.testng  An  IWorker  that is used to encapsulate and run Suite Runners   SuiteRunnerWorker(ISuite, SuiteRunnerMap, int, String)   - 
Constructor for class org.testng. SuiteRunnerWorker 
  
   SuiteRunState   - Class in  org.testng  A state object that records the status of the suite run.   SuiteRunState()   - 
Constructor for class org.testng. SuiteRunState 
  
   suiteThreadPoolSize   - 
Variable in class org.testng. CommandLineArgs 
  
 
 
    
 T  
 
   Test   - Annotation Type in  org.testng.annotations  Mark a class or a method as part of the test.   TEST_CLASS   - 
Static variable in class org.testng. CommandLineArgs 
  
   TEST_CLASSPATH   - 
Static variable in class org.testng. TestNG 
  
   TEST_JAR   - 
Static variable in class org.testng. CommandLineArgs 
  
   TEST_NAME   - 
Static variable in class org.testng. CommandLineArgs 
  
   TEST_NAMES   - 
Static variable in class org.testng. CommandLineArgs 
  
   TEST_RUNNER_FACTORY   - 
Static variable in class org.testng. CommandLineArgs 
  
   testClass   - 
Variable in class org.testng. CommandLineArgs 
  
   TestException   - Exception in  org.testng  Exception thrown when an exception happens while running a test
 method.   TestException(String)   - 
Constructor for exception org.testng. TestException 
  
   TestException(Throwable)   - 
Constructor for exception org.testng. TestException 
  
   TestException(String, Throwable)   - 
Constructor for exception org.testng. TestException 
  
   TestInstance   - Annotation Type in  org.testng.annotations  If this annotation is used on a parameter of a data provider, that parameter is the instance
 of the test method which is going to be fed by this data provider.   testJar   - 
Variable in class org.testng. CommandLineArgs 
  
   TestListenerAdapter   - Class in  org.testng  A simple ITestListener adapter that stores all the tests
 that were run.   TestListenerAdapter()   - 
Constructor for class org.testng. TestListenerAdapter 
  
   testName   - 
Variable in class org.testng. CommandLineArgs 
  
   testNames   - 
Variable in class org.testng. CommandLineArgs 
  
   TestNG   - Class in  org.testng  This class is the main entry point for running tests in the TestNG framework.   TestNG()   - 
Constructor for class org.testng. TestNG 
 Default constructor.
   TestNG(boolean)   - 
Constructor for class org.testng. TestNG 
 Used by maven2 to have 0 output of any kind come out
 of testng.
   TestNG.ExitCodeListener   - Class in  org.testng      TestNG.ExitCodeListener()   - 
Constructor for class org.testng. TestNG.ExitCodeListener 
  
   TestNG.ExitCodeListener(TestNG)   - 
Constructor for class org.testng. TestNG.ExitCodeListener 
  
   TestNGAntTask   - Class in  org.testng  TestNG settings:
 
 classfileset (inner)
 classfilesetref (attribute)
 xmlfileset (inner)
 xmlfilesetref (attribute)
 enableAssert (attribute)
 excludedGroups (attribute)
 groups (attribute)
 junit (attribute)
 listener (attribute)
 outputdir (attribute)
 parallel (attribute)
 reporter (attribute)
 sourcedir (attribute)
 sourcedirref (attribute)
 suitename (attribute)
 suiterunnerclass (attribute)
 target (attribute)
 testjar (attribute)
 testname (attribute)
 threadcount (attribute)
 dataproviderthreadcount (attribute)
 verbose (attribute)
 testrunfactory (attribute)
 configFailurepolicy (attribute)
 randomizeSuites (attribute)
 methodselectors (attribute)
 

 Ant settings:
 
 classpath (inner)
 classpathref (attribute)
 jvm (attribute)
 workingDir (attribute)
 env (inner)
 sysproperty (inner)
 propertyset (inner)
 jvmarg (inner)
 timeout (attribute)
 haltonfailure (attribute)
 onHaltTarget (attribute)
 failureProperty (attribute)
 haltonFSP (attribute)
 FSPproperty (attribute)
 haltonskipped (attribute)
 skippedProperty (attribute)
 testRunnerFactory (attribute)
 

 Debug information:
 
 dumpCommand (boolean)
 dumpEnv (boolean)
 dumpSys (boolean)
    TestNGAntTask()   - 
Constructor for class org.testng. TestNGAntTask 
  
   TestNGAntTask.Mode   - Enum in  org.testng      TestNGAntTask.TestNGLogSH   - Class in  org.testng      TestNGAntTask.TestNGLogSH(Task, int, int, boolean)   - 
Constructor for class org.testng. TestNGAntTask.TestNGLogSH 
  
   TestNGException   - Exception in  org.testng  The base class for all exceptions thrown by TestNG.   TestNGException(Throwable)   - 
Constructor for exception org.testng. TestNGException 
  
   TestNGException(String)   - 
Constructor for exception org.testng. TestNGException 
  
   TestNGException(String, Throwable)   - 
Constructor for exception org.testng. TestNGException 
  
   TestNGUtils   - Class in  org.testng      TestNGUtils()   - 
Constructor for class org.testng. TestNGUtils 
  
   TestRunner   - Class in  org.testng  This class takes care of running one Test.   TestRunner(IConfiguration, ISuite, XmlTest, String, IAnnotationFinder, boolean, List<IInvokedMethodListener>)   - 
Constructor for class org.testng. TestRunner 
  
   TestRunner(IConfiguration, ISuite, XmlTest, boolean, List<IInvokedMethodListener>)   - 
Constructor for class org.testng. TestRunner 
  
   testRunnerFactory   - 
Variable in class org.testng. CommandLineArgs 
  
   THREAD_COUNT   - 
Static variable in class org.testng. CommandLineArgs 
  
   threadCount   - 
Variable in class org.testng. CommandLineArgs 
  
   TimeBombSkipException   - Exception in  org.testng  A   SkipException   extension that transforms a skipped method
 into a failed method based on a time trigger.   TimeBombSkipException(String, Date)   - 
Constructor for exception org.testng. TimeBombSkipException 
 Creates a  TimeBombedSkipException  using the  expirationDate .
   TimeBombSkipException(String, Date, String)   - 
Constructor for exception org.testng. TimeBombSkipException 
 Creates a  TimeBombedSkipException  using the  expirationDate .
   TimeBombSkipException(String, String)   - 
Constructor for exception org.testng. TimeBombSkipException 
 Creates a  TimeBombedSkipException  using the  date 
 in the format  yyyy/MM/dd .
   TimeBombSkipException(String, String, String)   - 
Constructor for exception org.testng. TimeBombSkipException 
 Creates a  TimeBombedSkipException  using the  date 
 in the specified format  format .
   TimeBombSkipException(String, String, String, String)   - 
Constructor for exception org.testng. TimeBombSkipException 
 Creates a  TimeBombedSkipException  using the  date 
 in the specified format  inFormat .
   TimeBombSkipException(String, Date, Throwable)   - 
Constructor for exception org.testng. TimeBombSkipException 
 Creates a  TimeBombedSkipException  using the  expirationDate .
   TimeBombSkipException(String, Date, String, Throwable)   - 
Constructor for exception org.testng. TimeBombSkipException 
 Creates a  TimeBombedSkipException  using the  expirationDate .
   TimeBombSkipException(String, String, Throwable)   - 
Constructor for exception org.testng. TimeBombSkipException 
 Creates a  TimeBombedSkipException  using the  date 
 in the format  yyyy/MM/dd .
   TimeBombSkipException(String, String, String, Throwable)   - 
Constructor for exception org.testng. TimeBombSkipException 
 Creates a  TimeBombedSkipException  using the  date 
 in the specified format  format .
   TimeBombSkipException(String, String, String, String, Throwable)   - 
Constructor for exception org.testng. TimeBombSkipException 
 Creates a  TimeBombedSkipException  using the  date 
 in the specified format  inFormat .
   toString()   - 
Method in class org.testng. ReporterConfig 
  
   toString()   - 
Method in class org.testng. SuiteRunnerWorker 
  
   toString()   - 
Method in class org.testng. TestListenerAdapter 
  
   toString()   - 
Method in class org.testng.xml. XmlClass 
  
   toString()   - 
Method in class org.testng.xml. XmlSuite 
 
   toString()   - 
Method in class org.testng.xml. XmlTest 
  
   toXml(String)   - 
Method in class org.testng.xml. XmlClass 
  
   toXml(String)   - 
Method in class org.testng.xml. XmlInclude 
  
   toXml(String)   - 
Method in class org.testng.xml. XmlMethodSelector 
  
   toXml(String)   - 
Method in class org.testng.xml. XmlPackage 
  
   toXml()   - 
Method in class org.testng.xml. XmlSuite 
 Returns a String representation of this XML suite.
   toXml(String)   - 
Method in class org.testng.xml. XmlTest 
  
   transform(ITestAnnotation, Class, Constructor, Method)   - 
Method in interface org.testng. IAnnotationTransformer 
 This method will be invoked by TestNG to give you a chance
 to modify a TestNG annotation read from your test classes.
   transform(IConfigurationAnnotation, Class, Constructor, Method)   - 
Method in interface org.testng. IAnnotationTransformer2 
 Transform an IConfiguration annotation.
   transform(IDataProviderAnnotation, Method)   - 
Method in interface org.testng. IAnnotationTransformer2 
 Transform an IDataProvider annotation.
   transform(IFactoryAnnotation, Method)   - 
Method in interface org.testng. IAnnotationTransformer2 
 Transform an IFactory annotation.
 
 
    
 U  
 
   USE_DEFAULT_LISTENERS   - 
Static variable in class org.testng. CommandLineArgs 
  
   useDefaultListeners   - 
Variable in class org.testng. CommandLineArgs 
  
 
 
    
 V  
 
   validateCommandLineParameters(CommandLineArgs)   - 
Static method in class org.testng. TestNG 
 Double check that the command line parameters are valid.
   validateOptions()   - 
Method in class org.testng. TestNGAntTask 
  
   valueOf(String)   - 
Static method in enum org.testng. TestNGAntTask.Mode 
 Returns the enum constant of this type with the specified name.
   values()   - 
Static method in enum org.testng. TestNGAntTask.Mode 
 Returns an array containing the constants of this enum type, in
the order they are declared.
   VERBOSE   - 
Static variable in class org.testng. CommandLineArgs 
  
   verbose   - 
Variable in class org.testng. CommandLineArgs 
  
 
 
    
 W  
 
   wrapDataProvider(Class, Collection<Object[]>)   - 
Static method in class org.testng. ConversionUtils 
 Turns the output of a JUnit 4 @Parameters style data provider into
 one that is suitable for TestNG's @DataProvider.
 
 
    
 X  
 
   XML_PATH_IN_JAR   - 
Static variable in class org.testng. CommandLineArgs 
  
   XML_PATH_IN_JAR_DEFAULT   - 
Static variable in class org.testng. CommandLineArgs 
  
   XmlClass   - Class in  org.testng.xml  This class describes the tag   in testng.xml.   XmlClass()   - 
Constructor for class org.testng.xml. XmlClass 
  
   XmlClass(String)   - 
Constructor for class org.testng.xml. XmlClass 
  
   XmlClass(String, boolean)   - 
Constructor for class org.testng.xml. XmlClass 
  
   XmlClass(Class)   - 
Constructor for class org.testng.xml. XmlClass 
  
   XmlClass(Class, boolean)   - 
Constructor for class org.testng.xml. XmlClass 
  
   XmlClass(String, int)   - 
Constructor for class org.testng.xml. XmlClass 
  
   XmlClass(String, int, boolean)   - 
Constructor for class org.testng.xml. XmlClass 
  
   XmlInclude   - Class in  org.testng.xml      XmlInclude(String)   - 
Constructor for class org.testng.xml. XmlInclude 
  
   XmlInclude(String, int)   - 
Constructor for class org.testng.xml. XmlInclude 
  
   XmlInclude(String, List<Integer>, int)   - 
Constructor for class org.testng.xml. XmlInclude 
  
   XmlMethodSelector   - Class in  org.testng.xml  This class describes the tag    in testng.xml.   XmlMethodSelector()   - 
Constructor for class org.testng.xml. XmlMethodSelector 
  
   XmlPackage   - Class in  org.testng.xml  This class describes the tag    in testng.xml.   XmlPackage()   - 
Constructor for class org.testng.xml. XmlPackage 
  
   XmlPackage(String)   - 
Constructor for class org.testng.xml. XmlPackage 
  
   xmlPathInJar   - 
Variable in class org.testng. CommandLineArgs 
  
   XmlSuite   - Class in  org.testng.xml  This class describes the tag <suite> in testng.xml.   XmlSuite()   - 
Constructor for class org.testng.xml. XmlSuite 
  
   XmlTest   - Class in  org.testng.xml  This class describes the tag <test>  in testng.xml.   XmlTest(XmlSuite, int)   - 
Constructor for class org.testng.xml. XmlTest 
 Constructs a  XmlTest  and adds it to suite's list of tests.
   XmlTest(XmlSuite)   - 
Constructor for class org.testng.xml. XmlTest 
  
   XmlTest()   - 
Constructor for class org.testng.xml. XmlTest 
  
   XmlUtils   - Class in  org.testng.xml      XmlUtils()   - 
Constructor for class org.testng.xml. XmlUtils 
  
 
 
 A   B   C   D   E   F   G   H   I   J   L   M   N   O   P   R   S   T   U   V   W   X  

 
   
  
 
 
 
   
 
   
          Overview     
        Package   
        Class   
          Tree     
          Deprecated     
       Index    
          Help     
   
 
 
  
 
 
 

 
  
 PREV 
 NEXT  
  
    FRAMES     
   NO FRAMES     
  
    All Classes  ');
  }
  //-->
 
 
    All Classes
